# Immunological Responses to Envenomation

**DOI:** 10.3389/fimmu.2021.661082

**Published:** 2021-05-10

**Authors:** Rachael Y. M. Ryan, Jamie Seymour, Alex Loukas, J. Alejandro Lopez, Maria P. Ikonomopoulou, John J. Miles

**Affiliations:** ^1^ Division of Tropical Health and Medicine, Australian Institute of Tropical Health and Medicine, James Cook University, Cairns, QLD, Australia; ^2^ Centre for Molecular Therapeutics, Australian Institute of Tropical Health & Medicine, James Cook University, Cairns, QLD, Australia; ^3^ School of Environment and Sciences, Griffith University, Nathan, QLD, Australia; ^4^ QIMR Berghofer Medical Research Institute, The University of Queensland, Herston, QLD, Australia; ^5^ Translational Venomics Group, Madrid Institute for Advanced Studies (IMDEA) in Food, CEI UAM+CSIC, Madrid, Spain; ^6^ Institute for Molecular Bioscience, University of Queensland, Brisbane, QLD, Australia; ^7^ Centre for Tropical Bioinformatics and Molecular Biology, James Cook University, Cairns, QLD, Australia

**Keywords:** venom, detoxification, innate immunity, adaptive immunity, immunopathology, Irukandji syndrome, venom allergy, systemic inflammation

## Abstract

Venoms are complex mixtures of toxic compounds delivered by bite or sting. In humans, the consequences of envenomation range from self-limiting to lethal. Critical host defence against envenomation comprises innate and adaptive immune strategies targeted towards venom detection, neutralisation, detoxification, and symptom resolution. In some instances, venoms mediate immune dysregulation that contributes to symptom severity. This review details the involvement of immune cell subtypes and mediators, particularly of the dermis, in host resistance and venom-induced immunopathology. We further discuss established venom-associated immunopathology, including allergy and systemic inflammation, and investigate Irukandji syndrome as a potential systemic inflammatory response. Finally, this review characterises venom-derived compounds as a source of immune modulating drugs for treatment of disease.

## Introduction

Venoms are complex mixtures of proteins, peptides, biogenic amines, and salts produced by a diverse range of animals for predation, protection, and competition (1–4). In humans, needle-like stinging apparatuses inject venom compounds into dermal-epidermal junctions, capillary vessels, and skeletal muscle fibres (5). The consequences of envenomation range from innocuous to lethal (6, 7).

As the initial site of venom’s interaction with the immune system, the dermis provides protection through physical, chemical, and cellular defence mechanisms (8, 9). Prominent defenders in the dermal immune network include keratinocytes, endothelial cells, and tissue-resident and infiltrating immune cells for fast and non-specific responses (innate) and acquired long term protection (adaptive) (8, 9). The primary role of these cells is host defence. However, venom-mediated immune dysregulation can contribute to envenomation severity (10). Accordingly, this review discusses both the protective and pathological responses of barrier cells and the immune system towards venom compounds.

## Innate Responses to Envenomation

Defence against envenomation requires an acute response achieved by the body’s innate immune system. Innate mechanisms comprise barrier and cellular defences for immediate but non-specific resistance to foreign bodies (such as venom compounds), injuries, and pathogens. Physical barriers (skin and mucosal membranes) and secretions (chemical substances and enzymes) along with resident and infiltrating immune cells provide readily available protection without requiring prior exposure to the damaging compounds (11). Instead, sentinel and scavenger cells express receptors that sense evolutionarily conserved structures common to microbes, cellular stress, and harmful substances (12).

A wide diversity of innate signalling receptor and response types is responsible for efficient detection and neutralisation/elimination of various host threats (12). The detection of danger or stress signals initiates proinflammatory events. Broadly, these include the production of cytokines and chemokines for immune cell recruitment/activation, the release of antimicrobial peptides that directly kill pathogens, the phagocytosis and destruction of foreign particles and microbes, the generation of reactive oxygen species (ROS), reactive oxygen intermediates, and reactive nitrogen intermediates, and the release of enzymes with potent protein degrading and microbicidal properties (11).

Regulated innate effector functions are also critical for tissue repair and homeostasis (13). In addition, the presentation of foreign macromolecules, required for the establishment of acquired (adaptive) immune responses, is achieved by innate antigen-presenting cells (APC), including dendritic cells (DCs), monocytes (MNCs), and macrophages (MΦ) (11). Likewise, plasma proteins, including those of the complement system (an ancient protein defensive system), promote inflammation or directly kill pathogens (14).

Detection of venom compounds by innate mechanisms initiates inflammatory reactions critical to host protection, venom detoxification, and ultimately the resolution of symptoms (15, 16). Participation by the epidermis, endothelium, neutrophils, MNCs, MΦs, mast cells, and soluble effector mediators increases host resistance to the damaging events of bites and stings. Yet, as discussed below, many venom constituents can augment the activity of these components leading to venom-induced, immune-mediated host damage.

### Epidermis (Keratinocytes)

The epidermis, the outermost layer of the skin, comprises 95% keratinocytes arranged in four layers (17). Tight junctions formed by keratinocyte-derived proteins provide a physical barrier from the external environment and structural support for Langerhans cells (epidermal-resident DCs), melanocytes, Merkel cells (tactile epithelial cells), and sensory neurons (18). Keratinocytes serve important sentinel and proinflammatory functions, where cross-talk between keratinocytes and cells of the dermal-epidermal junction direct immune cell function and maturation during both initial and late phases of inflammation (19). Like cells of the immune system, keratinocytes express cytokine receptors and pattern-recognition receptors (PRRs), enabling the detection of pathogen-, damage-, and venom-associated molecular patterns (PAMPs, DAMPs, and VAMPS) (20, 21). Activation of keratinocyte proinflammatory genes, such as by venom compounds, initiates the synthesis and release of cytokines, nitric oxide (NO), and alarmins, stimulating resident immune cells and attracting immune cell infiltration (20, 21).

To counteract this defensive barrier, many animal venoms contain matrix metalloproteinase (MMP) and L-Amino acid oxidase (LAAO) enzymes (22–25). Venom-derived MMPs and LAAOs can induce keratinocyte cell death by autophagy, apoptosis, or necrosis (22–25). Proteolytic degradation of the dermis facilitates access of venom-derived toxins to the circulation, lymphatics, and target organs for prey/predator immobilisation (22–25). In some instances, the induction of apoptosis stimulates the overexpression of endogenous MMPs, indirectly triggering tissue destruction (26). For example, brown recluse spider (*Loxosceles rufescens*) bites cause significant dermonecrotic effects, systemic inflammation, and potentially death in children (26). Interestingly, the molecular mechanism underpinning the initiation of cutaneous necrosis (a common reaction in loxoscelism) involves keratinocyte-derived enzymes (26). Induction of apoptosis by *Loxosceles* sphingomyelinase D, the main component of *Loxosceles* venom, stimulates the expression/activation of secreted and membrane-bound MMP-2 and MMP-9 in keratinocyte cultures (26). It has been shown that the augmented expression of MMPs has a role in the necrotic skin lesions associated with *L. rufescens* envenoming (26). Hence, tetracycline has shown protective effect against venom-induced cell death by inhibiting the activation of MMP proenzyme precursors and MMP enzymatic activity (26).

### Endothelium

Throughout the vascular system, endothelial cells (ECs) line the interior surface of blood and lymphatic vessels (27). Although once considered bystanders in the inflammatory process, ECs can dictate inflammatory responses under homeostatic and pathophysiological settings (28). As a primary point of contact for bloodborne pathogens and other host assaults, including toxins, ECs play an important sentinel role (29–32). Expression of numerous PRRs, including toll-like receptors (TLRs) and receptors for tumour necrosis factor (TNF) and interleukin (IL)-1β, enables the intravascular detection of harmful compounds, the activation of proinflammatory genes, and the alteration of the microenvironment (29–32). ECs also express major histocompatibility complex (MHC) molecules, classes I and II, and costimulatory molecules, such as the CD40 ligand (CD40L) that allows intravascular antigen presentation and EC-mediated activation of effector memory T cells (T_EM_) (33). However, ECs principally modulate immune function by directing leukocyte trafficking and distribution (34–36). Leukocyte tethering, rolling, and extravasation occurs in response to highly selective expression of cell adhesion molecules (CAMs), such as intercellular adhesion molecule-1 (ICAM-1) and selectins, on the apical surface of ECs. CAMs are essential for the homing and migration of immune cells towards secondary lymphoid tissue and inflammatory foci (34–36). The powerful influence of ECs on immune function has led to hypothesise that immune dysregulation, such as seen in systemic inflammation, might be partially mediated by the endothelium (37). As venom compounds from different species can modulate EC function (described below), this may have important implications in venom-induced systemic inflammation or allergy.

Venom from different species can induce EC perturbations, including distortions in cellular function, morphology, cytoskeletal organisation, and cell viability (38–41). Collectively these actions alter vascular permeability and blood vessel stability (38–41). Additionally, snake and spider venoms are highly proinflammatory in EC cultures, commonly provoking the secretion of IL-6, IL-8, monocyte chemoattractant protein-1 (MCP-1/CCL2), and Regulated on Activation, Normal T Cell Expressed and Secreted (RANTES/CCL5) (42). Together, these events modify the extracellular environment and leukocyte activity in local and systemic compartments, which may have important implications for the pathology of some envenomations (41, 42). For instance, though neutrophil depletion abrogates *Loxosceles* venom-induced necrotic lesions, neutrophils are not a direct target of *Loxosceles* venom-derived toxins (43). While neutrophils are likely the proximal cause of inflammation and tissue destruction, direct exposure to venom does not provoke this response (43). Instead, research has shown that the venom contains EC agonists that elicit dysregulated activation and cellular damage (43). *Loxosceles* venom strongly stimulates EC-secretion of IL-8, a potent neutrophil chemoattractant, and low-level surface expression of E-selectin (43). Researchers have noticed an unusual activation response in neutrophils to these venom-mediated proinflammatory signals (43). Specifically, neutrophils adhere to venom-stimulated ECs *via* selectin-mediated tethering in a time- and dose-dependent manner, yet without transmigration (43). In culture, these sequestered leukocytes rapidly increase intracellular Ca^2+^ levels and release primary and secondary granules containing the lytic enzymes responsible for tissue degradation (43). Accordingly, the initiation of *Loxosceles* necrotic lesions appears to be dependent upon toxin-mediated EC responses (43). These findings, further to work by Paixão-Cavalcante and colleagues, suggest a role for immune-targeted (in addition to toxin-targeted) therapeutic strategies for envenomation (26).

### Mononuclear Phagocytic System

MNCs and MΦs form a crucial phagocytic component of innate immunity. Both MNCs and MΦs are highly migratory, enabling tissue surveillance, antigen capture, and migration to draining lymph nodes for antigen presentation to adaptive immune cells (44, 45). As such, they function primarily as sentinel phagocytes and regulators of immunity (46). MNCs and MΦs secrete a wide range of cytokines and chemokines that modulate immune cell function and are potent mediators of neutrophil recruitment (47).

Many animal venoms can modulate the metabolism and function of MNCs and MΦs. For example, *Crotalus durissus terrificus* venom (CDTV) significantly inhibits the trafficking and phagocytic capacity of rat peritoneal-resident, thioglycollate‐elicited, and *Mycobacterium bovis* strain bacille Calmette Guérin (BCG)-activated MΦs, without affecting cell viability at 2 h, 4 days, or 7 days post intraperitoneal administration (48). In contrast to these immunosuppressive effects, CDTV can enhance the production of hydrogen peroxide (H_2_O_2_) and NO from phorbol 12-myristate 13-acetate-stimulated resident, elicited, and activated MΦs (48). Further, CDTV-treatment augments cellular metabolism *ex vivo*. Extracted peritoneal cells showed upregulated glucose and glutamine usage and increased maximal activity of hexokinase, glucose‐6‐phosphate dehydrogenase, citrate synthase, and phosphate‐dependent glutaminase (48). These venom-mediated actions result in amplified MΦs candidacidal activity and decreased phagocytosis potential (48).

Comparably, venom from the pit viper, *Bothrops alternatus* (BAV), stimulates increased production of superoxide anion $\left(O_{2}^{-}\right)$ from isolated thioglycollate‐elicited MΦs (49). Again, BAV-treatment showed a limited impact on MΦ viability, as evaluated by Trypan blue exclusion, and did not interfere with MΦ’s adhesion or detachment capacity up to 100 µg/mL BAV (49). Pretreatment with the protein kinase C inhibitor, staurosporine (14 nM/mL), suppressed $O_{2}^{-}$ production and phagocytosis, suggesting the involvement of a PKC-dependent signalling pathway (49). However, unlike CDTV, Setubal et al. observed increased MΦ complement receptor (CR3)-mediated phagocytosis following incubation with BAV (49). Phagocytosis of serum-opsonised zymosan particles was significantly higher in venom-stimulated MΦs compared to vehicle control (49). It was hypothesised that increased phagocytic activity and excessive release of superoxide might be involved in the local tissue destruction caused by *B. alternatus* snakebite (49).

Studies using human MNCs have revealed the potent proinflammatory properties of different venom compounds. For example, venom from the *Androctonus crassicauda* scorpion induces IL-12p40 mRNA expression and protein secretion from purified MNCs (50). However, venom exposure also produced concentration- and time-dependent cytotoxicity, as evidenced by significant LDH release in MNC cultures (50). Further examples include a C-type lectin (BjcuL) isolated from *Bothrops jararacussu* snake venom that induces TNF production from resting CD14^+^ cells without stimulating proliferation (51). Phospholipase D from *Loxosceles laeta* spider venom promotes MNC migration in THP-1 cell cultures and cytokine release from skin fibroblasts (52). *Bothrops* snake venoms provoke the release of proinflammatory mediators, prostaglandin E_2_ (PGE_2_), macrophage inflammatory protein 1-alpha (MIP-1α/CCL3), and IL-1β, and induces activation of NF-κB in human MNCs (53). Given the immunostimulatory role of MNCs and MΦs on immune function, these data demonstrate the capacity of venom to induce systemic inflammatory responses.

Contrasting this research, Khemili et al. examined the immunosuppressive potential of ion channel modulators from scorpion venom using murine MΦs (54). Voltage-gated potassium channels (K_V_) play a crucial role in calcium signalling and immune cell excitability (54). In the resting state, murine MΦs predominantly express the K_V_1.5 subunit of the K_V_1.5/K_V_1.3 heterotetrameric complex (54). Innate activation, including LPS stimulation, induces K_V_1.3 overexpression (54). Using non-cytotoxic concentrations of *Androctonus australis hector* (Aah) venom, the authors observed a voltage-independent inhibition of K_V_ current amplitude in LPS-activated (M1) MΦs (54). On the contrary, venom perfusion did not significantly decrease K_V_ current amplitude in resting cells (54). These results suggest the presence of an ion channel blocker with a higher affinity for the K_V_1.3 subunit, abundant on the cell surface of activated MΦs (54). However, as indicated by the authors, the downstream functional consequences of MΦ ion channel modulation requires further examination (54).

Additional immunomodulatory functions, such as TLR inhibition, have been identified using synthetic venom components (55). TLR signalling is a critical element in innate detection and MΦ activation (56). Many animal venoms contain VAMPs that strongly provoke immune stimulation *via* TLR engagement (56). Contrasting this, recombinant rhodostomin (Rn), a snake venom-derived disintegrin, exhibits potent TLR2 inhibition against lipopeptide-stimulated THP-1 cells (55). Incubation with Rn suppresses TNF, IL-1β, and IL-8 release and IκB degradation from Pam3CysSerLys4-activated cells (a TLR1/TLR2 agonist), in a dose-dependent manner (55). In THP-1 cell cultures, Rn reverses the phosphorylation of focal adhesion kinase downstream kinases, thereby inhibiting signal transduction (55). In the caecal ligation and puncture (CLP) model of sepsis, Rn significantly suppresses CLP-induced TNF, IL-6, and MCP-1 production and reduces animal mortality (55). Histology has also revealed that Rn significantly alleviates CLP-induced tissue-damage (55). Studies such as these highlight venom as a source of compounds for drug discovery.

### Granulocytes (Neutrophils)

Neutrophils are the most abundant leukocyte, constituting 40-75% of circulating white blood cells (WBC) (57). Derived from pluripotent stem cells in the bone marrow, a segmented nucleus of three to five lobes and the presence of secretory vesicles/granules characterise mature cells (58). Although short-lived, estimates range from hours to several days, they are the first phagocyte recruited and mobilised from the bone marrow or periphery to the infection/injury (59, 60). Upon arrival, these granulocytes directly destroy pathogens, inactivate toxins, and mount inflammatory responses through oxidative and non-oxidative pathways (59, 60). Like other immune cells, neutrophils are prolific producers of cytokines and chemokines and can mount robust proinflammatory responses (61).

For non-infectious/sterile challenges, such as envenomation, exocytosis of granules/secretory vesicles releases up to 700 defensive proteins into the extracellular milieu (58). These proteins include defensins, serine proteases, neutrophil elastase, proteinase 3, cathepsin G, cytokines, and chemokines, some of which inactivate venom components through proteolytic degradation (62). An additional neutrophil defensive strategy, critical during envenomation, is the neutrophil extracellular trap or “NET”. NET formation (NETosis) occurs through programmed self-destruction, whereby the release of nuclear DNA forms a sticky “net” of extracellular fibres, containing the dissemination of toxins, bacteria, and pathogens (63, 64). However, whether neutrophils protect against or promote venom injury is disputed. Certainly, the participation of neutrophils in venom-associated pathologies, such as dermonecrosis, has been well documented (26, 43). Nevertheless, neutrophilic functions, including toxin trapping and inactivation, provide critical defence against systemic injury and death (65). Additionally, neutrophil clearance of necrotic tissue is essential for muscle regeneration following snakebite (16).

Snake venom, such as from *Echis carinatus*, induces NETosis and ROS generation in a time- and dose-dependent manner in animal models and cell cultures (65). While these neutrophilic-defensive actions hinder venom’s systemic dissemination, dense NET accumulation can block blood vessels, resulting in localised tissue damage and impeding antivenom’s efficacy (65). Unfortunately, though research shows that co-treatment with DNase 1 prevents tail injury in *E. carinatus* experimentally envenomed rodents, mortality is significantly higher among these mice (65). Interestingly, follow-up work by Stackowicz et al. determined that localised tissue damage is neutrophil independent (66). Despite verifying that DNase-treatment does indeed reduce tail injury at the expense of survival, the study reported similar occurrences in both neutrophil-sufficient and deficient settings (66). These data suggest that extracellular DNA from multiple dying cell types, including neutrophils, mediate capillary obstruction following envenomation.

Regardless of DNA source, toxin retention inhibits systemic injury to the detriment of the localised compartments (66). NET formation and capillary obstruction can lead to severe consequences, such as amputation, which has devastating implications for victims’ lives (67). Accordingly, there is an urgent need for effective therapeutics that minimise tissue necrosis and facilitate antivenom efficacy. However, given that neutrophil participation is critical in tissue repair post-envenomation, neutrophil-targeting therapies may be counterproductive (16). Hence, further research is required.

### Granulocytes (Mast Cells)

Mast cells (MCs) are long-lived, tissue-resident effector cells derived from a myeloid lineage and matured under the influence of stem cell factor and cytokines (68). MCs are positioned near entry points of mucosal, epithelial, and sub-endothelial connective tissue to provide innate defence and perform a wide range of physiological functions that maintain tissue homeostasis (68). MCs induce killing and assist in the clearance of parasites and pathogens. For venom/toxin defence, sequestering and neutralisation occur (69). Expression of multiple PRRs on the cell surface enables rapid detection and response to immune challenges, including venom toxins. Activation of PRRs induces *de novo* synthesis of cytokines, chemokines, and eicosanoids to attract and stimulate other effector cells (70). A classic feature of MCs are weaponised granules, containing preformed toxic inflammatory mediators, including enzymes (tryptase, chymase, and carboxypeptidase A3), amines (histamine and heparin), and cytokines (TNF). MC activation, mediated by immunoglobulin E (IgE)-bound FcϵRI, causes rapid degranulation potentially inducing a systemic proinflammatory response (71, 72).

Despite the widely recognised role of MCs in allergy and anaphylactic shock, animal models have provided evidence of MCs’ protective function against envenomation (73, 74). As reviewed by Galli et al., functional MCs enhance the survival of mice challenged with sub-lethal doses of snake (*Atractaspis engaddensis; Daboia russelii)*, Gila monster *(Heloderma suspectum)*, European honey bee *(Apis mellifera)*, and scorpion (*Leiurus quinquestriatus hebraeus; Centruroides exilicauda)* venoms (75). The significantly higher mortality among MC-deficient mice has been attributed to the dysregulation of serine proteases (carboxypeptidase A3 and mast cell protease 4), which degrade peptides, and heparin and histamine (69, 75–77). In healthy individuals, the release of heparin and histamine can neutralise the effects of venom-derived toxins (69, 71). Adversely, the release of these amines provokes dangerous allergic symptoms in hypersensitive individuals, particularly in response to Hymenopteran venom (the venom of bees, wasps, and ants) (70).

### Chemical Mediators

The immune network is vast and highly complex. Intercellular communication across the network requires small soluble protein effectors, known as cytokines (78). Cytokines (interferons, interleukins, chemokines, and growth factors) are secreted by cells to instruct and regulate the immune system’s activity for protection against injury, infection, and disease (78). Biological functions include cellular activation, proliferation, differentiation, growth, and immune regulation (78). Further, as chemoattractant proteins, chemokines exert their effects *via* cell recruitment, migration, and adhesion (79). Like hormones, cytokines have autocrine, paracrine, or endocrine functions for localised or systemic effects (78). Broadly, they elicit either pro or anti-inflammatory action (80, 81). The reality, of course, is more complicated as many cytokines exhibit pleiotropic effects that are dependent on cellular source, target receptor, and the stage of the inflammatory process (80, 81). Additionally, immune cells adapt to the overall profile of the cytokine milieu they encounter (80, 81).

The expression and release of these potent chemical mediators are tightly regulated (82, 83). Nevertheless, infection, cancer, injury, disease (such as autoimmunity), and medical interventions (including drugs and organ transplant) can provoke dysregulation in cytokine levels resulting in devastating pathophysiological effects. Unchecked, cytokines and other proinflammatory mediators cause severe tissue destruction, systemic pathology, multiple organ failure, and potentially death (82, 83). Existing literature extensively describes diverse pathophysiology induced by dysregulated inflammatory mediators. These include cytokines (IL-1β, IL-6, TNF, IFN-γ, IL-10, IL-12, and GM-CSF) and chemokines (IL-8, MCP-1, eotaxin/CCL11, IP-10/CXCL10, MDC/CCL22, MIP-1α, and TARC/CCL17), as well as bradykinin, eicosanoids (prostaglandins and leukotrienes), cyclooxygenases, NO, and histamine (84). Dysregulation of these mediators is associated with inflammatory and neuropathic pain (85, 86), tissue destruction (87), systemic inflammation (88–90), autoimmunity, and allergic reactions (91). Unsurprisingly, the same proteins are detected in the serum of victims of envenomation, where pain and systemic injury occur (92, 93). Notably, similar secretion profiles are also present in experimentally envenomed animals and cell cultures (92). Additionally, venoms can have detrimental effects on platelet function and components of the complement system (94, 95). In particular, snake venoms can trigger critical pathologies, such as venom-induced consumption, thrombocytopenia, and hemorrhage (94, 95).

Cytokines and their respective receptors represent important immunotherapeutic targets for numerous conditions (96). Accordingly, it might seem plausible that targeting proinflammatory cytokines, chemokines, and small molecules (or their receptors) similarly represents novel therapeutic avenues for certain envenomations. However, research in this area is still in its infancy, and to date, studies have described both beneficial and detrimental outcomes of immunosuppression during experimental envenomation. For example, the detection of snake and bee venom toxins by NOD-like receptor family, pyrin domain-containing 3 (NLRP3) inflammasome, triggers immune cell activation, potent IL-1β secretion, and neutrophil influx (15). Interestingly, Palm and Medzhitov showed that although inflammasome inhibition, such as seen in caspase-1-deficient mice, successfully inhibited cytokine release and leukocyte influx, it unexpectedly resulted in a higher susceptibility of the mice to the noxious effects of venoms, including mortality (15). Conversely, Zoccal et al. determined that using a hexapeptide ligand for the MΦ scavenger receptor (CD36) protected mice against a lethal dose of *T. serrulatus* scorpion venom through decreased production of IL-1β, IL-6, TNF, CCL3, and PGE2, and restrained lung inflammation (97). While reduced IL-1β secretion and neutrophil influx was observed in both models, together, these data demonstrate the importance of innate immunodetection in the defence against bites and stings.

### Adaptive Responses to Envenomation

The immune system’s adaptive arm is predominantly comprised of B cells and T cells. The primary effector function of B cells is the generation of antibodies (immunoglobulins; Ig) for humoral defence (98). T cell effector functions are produced by a range of subsets, including cytotoxic (CD8^+^) T cells and helper (CD4^+^) T cells (TH) cytotoxic (CD8^+^) and helper (CD4^+^) lymphocytes. Cytotoxic CD8^+^ T cells protect against intracellular pathogens and suppress infectious disease and tumour growth, while CD4^+^ T cells maintain homeostasis and shape proinflammatory and regulatory immune responses (99).

Bites, stings, and intentional venom inoculation stimulate the generation of venom protein-specific antibodies (100, 101). Antibody-mediated neutralisation effectively counteracts venom activity (102). However, a primary B cell response is slow (requiring days to weeks to become fully active), while defence against rapid venom action requires an immediate response (103). As an alternative to host antibodies, antivenom, produced in large mammals and purified for medical purposes, can provide passive immunity to victims of life-threatening envenomation (104–106).

The following provides a simplified overview of a primary (thymus-dependent) humoral response towards envenomation. Following bite or sting, APCs, such as DCs, MNCs and MΦs, capture and process venom proteins at the site of injury, promoting maturation (100, 101). Matured APCs migrate to secondary lymphoid tissue to present venom antigen to naïve T_H_ cells *via* membrane-bound peptide-MHC II protein complexes (100, 101). In lymph nodes, engagement of a T cell receptor (TCR) with cognate peptide-MHC molecule initiates T_H_ activation (signal 1) (107). Critical secondary signals, required for complete T cell activation, are provided by APCs. APCs, especially DCs, highly express ligands (including CD80 and CD86) for T cell co-stimulatory molecules, such as CD28 (signal 2) (108). Next, APC-derived and circulating cytokines (as well as autocrine IL-2) induce T cell proliferation and differentiation (signal 3). For extracellular immune challenges, such as envenomation, CD4^+^ T cells acquire a T_H_2 phenotype with effector functions that include B cell activation (100, 101).

During a primary antibody response, B cells require multiple stimulatory signals. The first occurs when a B cell receptor (BCR) encounters its specific soluble or membrane-bound epitope (100, 101). The internalised antigen is processed and displayed on the B cell surface as a peptide-MHC complex for T_H_ presentation (100, 101). TCR binding triggers upregulation of co-stimulatory ligands, such as CD40L, and the production of proinflammatory cytokines, including IL-4 (107). CD40L engagement with B cell CD40 mediates the recruitment of intracellular adaptor proteins essential for propagating downstream signalling (107). Additionally, cytokines secreted by primed T_H_2 cells provide B cells with accessory stimulation for the early (proliferation and clonal expansion) and later (differentiation, antibody production, and isotype switching) stages of B cell activation (109). Proliferating B cells form germinal centres (GCs) where memory B and antibody-secreting plasma cells develop. Here, B cells also undergo somatic hypermutation and isotype switching (IgM and IgD to IgG, IgE, or IgA) to generate high-affinity antibodies for robust immune responses (110).

Yet, critical though they may be, adaptive responses can also produce severe pathology (74). For example, IgE isotype switching following venom challenge can, in a percentage of hypersensitive individuals, lead to fatal allergic reactions (discussed below) (74). In addition to allergy, dysregulation of adaptive responses and loss of self-tolerance stimulate destructive auto-reactivity (111). As such, lymphocytes (T cells in particular) are a target for therapeutic modulation (111). Serendipitously, venoms can contain ligands for T cell ion channels and receptors, able to modulate immune function with high specificity (described below) (112–121).

## Venom-Induced Immunopathology

Cell-specific venom-mediated immune dysregulation is described above. The following sections discuss modes of immunopathology, including venom-induced allergic reaction and systemic inflammation.

### Venom Allergy

Despite a notorious reputation for venomous snakes, spiders, and jellyfish, Australia’s largest proportion of venom-related fatalities occur due to anaphylactic events (122). Reflecting a global trend, honey bee (*A. mellifera*) stings are a significant contributor to venom injury in Australia, representing 16.3% of anaphylactic fatalities between 1997 and 2013 (123).

Venom from stinging Hymenopterans is commonly associated with allergic reactions worldwide (123). While most sting responses are localised and self-limiting, fatality can occur due to immune-mediated respiratory and/or cardiovascular failure (124). In these incidences, systemic reactions (SR) are predominantly mediated by IgE-mechanisms; however, dose-dependent IgE-independent responses are also possible (7).

Among venom-sensitised individuals, SR’s develop in 0.3% to 8.9% of cases (124, 125). Accordingly, Hymenopteran major allergens (antigens that bind IgE in greater than 50% of venom-sensitive individuals) have been well-characterised (7). For honey bee venom, these hypersensitivity-inducing proteins include phospholipase A2, hyaluronidase, acid phosphatase, and dipeptidylpeptidase (124). In vespid venom (wasp and yellow jacket), Antigen 5 and phospholipase A1 are the recognised major allergens (124).

Classic IgE-mediated allergic disease begins with a sensitisation process. Keratinocytes and resident immune cells detect damage induced by noxious substances, such as venom-derived compounds, stimulating the release of alarmins, cytokines (IL-4, IL-5, and IL-13), and other proinflammatory mediators required for antibody production (126, 127). DCs capture and process antigen for presentation to naïve T cells in draining lymph nodes, triggering events eventuating in plasma cell IgE antibody production (126, 127). Elevated IgE is a normal physiological response following a bite or sting and is not necessarily predictive of disease (69, 75, 124). Nevertheless, in some individuals, systemic IgE levels remain elevated longer term and can trigger SR, including anaphylactic shock, after multiple stings (7).

The symptoms of immediate (Type-1) allergic reactions occur during secondary antigen challenges. When IgE encounters its cognate antigen, crosslinking of FcϵR1 on MCs and basophils in mucosal and epithelial tissues provoke activation and degranulation (128–130). Preformed inflammatory mediators, including histamine and proteases, are rapidly released from granules into the extracellular environment (128–130).

Histamine is chiefly responsible for the clinical consequences of Type-1 allergic reactions (131, 132). Histamine’s protective functions include toxin binding and deactivation (69, 75). In allergic disease, histamine (acting upon H_1_ and H_2_ receptors) causes smooth muscle contraction, constriction of airways, swelling of the epiglottis, and increased vascular permeability. These events lead to dangerously low blood pressure, oedema and potentially death (131, 132). Further to this, proinflammatory genes, stimulated during the initial phase, induces *de novo* synthesis of the leukotrienes (particularly LTB_4_), cytokines, and chemokines responsible for the late phase (or delayed-type) symptoms (133, 134). These mediators are potent inducers of cell activation, migration, and the influx of lymphocytes and neutrophils (128, 134). The incidence and severity of biphasic anaphylaxis are highly variable, and fatalities can occur, necessitating continued patient observation following the resolution of initial symptoms (132, 133, 135).

The acute nature of fatal anaphylactic shock means death is more likely to occur in the home (87% of cases) than in the hospital (122, 123). Adrenaline autoinjectors (AAI) are an essential first-line treatment; however, death may still occur despite prompt administration (123, 136). Additional therapies include H_1_ and H_2_ antihistamines to counter the pathophysiological effects mediated through these receptors and critical supportive care (137, 138). For individuals with verified IgE-mediated allergy, venom immunotherapy (VIT) may generate a lifesaving tolerance to known allergens (139).

The pathogenic role of MCs and IgE-mediated granule release is well established (74). However, it has been postulated that allergy may be a barrier function disease in which cellular damage and perturbations of the epithelium and endothelium induce excessive proinflammatory responses from the resident immune cells (140). If this hypothesis is correct, the therapeutic modulation of these cells may correct the imbalanced proinflammatory response but this hypothesis has not been investigated for venom-associated allergy.

### Hypersensitivity to Marine Stings

In Australia, contact with venomous marine animals and plants accounts for 9% of venom-related hospitalisations (122). The phylum *Cnidaria* (classes Hydrozoa, Scyphozoa, and Cubozoa) comprises approximately 10,000 jellyfish species distributed throughout the world (138, 141). Of these, ~1% are medically relevant (142). Jellyfish stings typically trigger local or large local responses, manifesting as pain, swelling, and erythema, but are usually not life-threatening (143, 144). However, severe delayed cutaneous reaction, allergy, and anaphylactic shock can occur (145–147).

It may not be surprising then that in 1902 the unexpected discovery of anaphylaxis by physiologists Charles Richet and Paul Portier involved marine venom from the Portuguese man-of-war (*Physalia physalis*) and sea anemone (148, 149). When attempting to immunize dogs against harmful venom effects, Richet and Portier found that rather than confer protection (phylaxis), a subsequent venom challenge resulted in death. Although allergy was yet to be characterised, the experiments recognised immune involvement and the term “anaphylaxis” (against protection) was coined. This discovery later won Richet the 1913 Nobel Prize in Physiology or Medicine (148, 149).

Jellyfish envenomation is the most common marine sting type, impacting fishers, surfers, and sea bathers globally. An estimated 150 million stings occur annually, with peak incidence coinciding with blooming (or swarming) seasons during warmer months (148). A characteristic feature of the phylum *Cnidaria* is specialised stinging organelles, known as nematocysts. Located within cnidocytes, nematocysts are explosive capsule organelles containing coiled, barbed and threadlike tubules coated in venom (150). High-velocity capsule release, triggered by physicochemical stimuli, causes inversion of tubules into harpoon-like threads able to puncture and penetrate prey and predators (151, 152). Through nematocysts, jellyfish venom, containing pore-forming compounds, metalloproteases, serine proteases, and phospholipases, is injected into the victim, causing paralysis of prey and in humans dysregulation of immune function, cardiac function, respiratory function, and potentially fatal outcomes (153).

Although jellyfish venom can cause severe immediate-phase and delayed-type allergic reactions, the causative allergens are mostly unknown. *Chironex yamaguchii* is the box jellyfish species responsible for 78% of reported stings in Japan (144). Recently, the N-linked glycoprotein, CqTX-A (a hemolytic toxin), was identified as a major allergen from this venom (144). While the underlying mechanism has yet to be elucidated, this finding has important implications as CqTX-A shares significant sequence homology with other lethal pore-forming jellyfish proteins, specifically, CfTX-1 and CfTX-2 (*Chironex fleckeri*), CrTX-A (*Carybdea rastoni*), CaTX-A (*Carybdea alata*) and CqTX-A (*Chiropsalmus quadrigatus Haeckel*) (154–157).

For Hydrozoa (which includes Portuguese man-of-war), Scyphozoa (true jellyfish), and Cubozoa (box jellyfish), nematocysts are located on the tentacles, oral arms, and in some instances, the bell of the jellyfish (158, 159). Contact with tentacles can result in inoculation from potentially millions of nematocysts (160). Application of acetic acid and careful removal of tentacles from the victim’s skin prevents the further discharge of unfired nematocysts but does not deactivate the already injected toxins (161). Once stung, venom distribution occurs *via* capillaries and the lymphatic system to target organs, while the barbed tubules remain embedded in the skin until clearance (149, 160). Tubules are allergenic scaffolds comprising carbohydrates, proteins, chitin, and mini-collagen (148, 149, 162–164). As such, it has been suggested that impaired clearance, especially of chitin, may contribute to more severe outcomes of envenomation and hypersensitivity (149).

Beyond stings, there are recently described cases of severe allergic reactions to edible jellyfish consumption (147). It has also been reported that jellyfish stings, particularly among surfers, lead to sensitisation of foods containing gamma-glutamic acid, such as fermented soybean (165, 166). Collectively, these data highlight the antigenic properties within jellyfish nematocysts and tissue, in addition to their venom-derived destructive potential.

### Systemic Inflammation

Systemic inflammation, including cytokine release syndrome (CRS), is a life-threatening immune condition triggered in response to endotoxemia, severe viral infections (including influenza), and immunotherapies (167–169). Clinical manifestations can include fever, nausea, tachycardia, dyspnea, headache, muscle and joint pain, and in severe cases, neurotoxicity, pulmonary oedema, respiratory failure, and death (167). Certain envenomations can similarly provoke a systemic inflammatory response, most notably is scorpionism (10).

Scorpion envenomation is another leading cause of venom-associated morbidity and mortality, affecting more than one million individuals per annum (6). Although most stings produce only local symptoms (81% of cases), envenoming by dangerous species can initiate a surge of endogenous neurotransmitters, adrenaline and noradrenaline, resulting in an autonomic storm and severe systemic effects (6, 10). Additionally, venom-derived toxins induce spontaneous acetylcholine (Ach) release from peripheral nerves, responsible for the life-threatening cardiac dysfunction seen in severe cases (170). Interestingly, these mediators are also implicated in the box jellyfish pathology, Irukandji syndrome (171).

Along with intense acute pain and distress, severe scorpion envenoming (grade III stings) produces complex pathophysiology in victims (172). Like CRS, symptoms can include respiratory distress, cardiac dysfunction, pulmonary oedema, multiple organ failure, and potentially death, especially among children and the elderly (173). These clinical consequences are principally mediated by neurotoxic peptides, able to cause hyperexcitability of the autonomic nervous system through Na^+^, K^+^, Ca^+^ or Cl^−^ ion channel modulation (172). Ion channel modulation is also implicated in the development of pulmonary oedema, a symptom present in many fatal sting cases (174). Research led by Comellas et al. observed decreased lung fluid clearance in *Tityus serrulatus* envenomed rats, postulating venom-induced impairment of Na^+^/$K_{-}^{+}$ ATPase in alveolar epithelial cells as the mechanism (174).

Beyond neurotoxic effect, the immune network plays a role in significant scorpion envenomations (175). Immune participation in SR is multifactorial, involving direct antigenic activation and indirect stimulation *via* the neuroendocrine-immune axis (176). In addition to neurotransmitters, stings induce a rapid release of proinflammatory mediators. Elevated IFN-γ, IL-1β, IL-6, IL-8, IL-10, and TNF have been detected in the plasma of sting patients and animal models of scorpionism (10). Scorpion venom also provokes hypersensitivity mediators, particularly histamine (177). Blockade of the histamine H1 receptor has shown to be protective in *Androctonus australis hector* envenomed mice (177). Specifically, pretreatment with hydroxyzine (H1 receptor antagonist) reduced immune cell infiltrate and oedema in the brain and spinal cord and diminished levels of circulating proinflammatory cytokines (177). Further, scorpion toxins activate components of the complement system, including the generation of anaphylatoxins, which are potent chemotactic proteins (10). As further evidence of immunological involvement, heightened dermal reactions are reported in individuals predisposed to scorpion venom, such as seen in delayed-type hypersensitivity reactions (10).

For direct immunological activation, the most extensively studied species is the Brazilian scorpion, *T. serrulatus* (10). Whole *T. serrulatus* venom (TsV) and select purified toxins are potent stimulators of innate immune cells, including MΦs (56). *In vitro* assays have revealed that surface receptors TLR2, TLR4, CD14, and CD36 recognise TsV compounds, triggering cellular activation and production of cytokines and lipid mediators (56). Engagement of CD14 and co-receptor TLR4 promotes NF-κB and AP-1 signalling pathways and transcription of potent proinflammatory genes, including IL-1β (10). Consequently, TsV stimulates cytokine release from innate immune cells in a time- and dose-dependent manner, independent from cytotoxic effect (10). In addition, NF-κB signalling regulates cyclooxygenase-2 (COX-2) expression and the secretion of eicosanoid, PGE_2_ (178). PGE_2_ is a lipid mediator with pleiotropic roles in the initiation and resolution of inflammation, particularly inflammatory pain (179). Among its diverse biological functions, PGE_2_ activates IL-1β, MCP-1, and IL-6 pathways *via* prostaglandin EP4 receptor signalling (179). Accordingly, IL-1β and its receptor (IL-1R) are strongly suppressed by EP4 antagonism (179). IL-1β and IL-1R are potential therapeutic targets for multiple inflammatory diseases, including scorpion envenomation (180). As such, inhibition of the COX-2/PGE_2_/EP4 pathway has shown a cardiopulmonary protective effect in envenomed mice (97, 170).

The eicosanoid leukotriene B4 (LTB_4_) is also upregulated in cell culture and animal plasma following treatment with whole TsV or purified toxins (178). A study by Zoccal et al. demonstrated that activation of the class B scavenger receptor, CD36, directs eicosanoid metabolism towards LTB_4_ *via* a 5-lipoxygenase (5-LOX)/peroxisome proliferator-activated receptor gamma (PPAR-γ) pathway, opposing the events of TLR and CD14 receptor signalling (97). CD14 and TLR4 appear to be critical for TsV-induced cytokine and eicosanoid secretion (180). Further work by Zoccal and colleagues showed that CD14^-/-^ mice fail to produce significant levels of PGE_2_ or IL-1β post-TsV envenomation (180). In addition, CD36^obl/obl^ mice secrete increased levels of PGE_2_ and IL-1β post-TsV envenomation but do not produce LTB_4_ (180). Critically, LTB_4_ synthesis suppresses IL-1β maturation and secretion and the associated animal mortality (178). CD36, therefore, represents a novel therapeutic target for severe scorpion envenomation (180).

Mouse models of TsV envenomation produce autonomic dysfunction that is similar to clinically observed symptoms (170). A lethal inoculation of TsV induces sweating, ocular and nasal secretions, lethargy, and convulsions in mice, preceding cardiovascular disturbances and death (170). Observed hyperglycemia and neutrophilia are also consistent with sting patients (170). The neurotransmitters adrenaline and ACh, responsible for sympathetic and parasympathetic symptoms, respectively, are elevated in peripheral blood as well as in cardiac tissue in response to TsV (170). Treatment with atropine, a muscarinic receptor antagonist, but not propranolol, prevented venom-induced cardiovascular alterations, which are a leading cause of death in severe scorpionism (170). Curiously, despite showing systemic elevation of adrenaline, the study did not investigate the effect of an alpha-adrenergic blocking agent, such as prazosin (170).

In parallel to excessive ACh, lethal TsV envenomation stimulates the systemic and cardiac secretion of PGE_2_ and IL-1β. Reis et al. have recently proposed IL-1R as a neuro-immune link responsible for innate heart inflammation and TsV-induced heart failure (170). Research by their group demonstrated that TsV co-administered with PGE_2_ enhanced IL-1β and ACh release from cardio fibroblasts, an effect which was blocked by an EP receptor antagonist. In contrast, IL-1R silencing repressed PGE_2_, IL-1β and ACh levels and rescued mice from fatal TsV administration (170). As such, the study determined that PGE_2_ amplifies IL-1β release, which upon binding IL-1R potentiates upregulation of PGE_2_ and PGE_2_-dependent ACh release post TsV envenomation (170).

Existing scorpion sting management comprises specific antiserum and symptomatic treatment, such as pain and low dose anti-inflammatory medications (6). Grade III stings and stings in children younger than 15 require intensive care (6). Polymorphism within scorpion venom-derived proteins is geographically varied, impeding the manufacture of a standardised antivenom (6). Variability in toxin immunogenicity further limits the usefulness and cost-effectiveness of antivenom production (6). Accordingly, some experts challenge the use of antiserum therapy due to insufficient neutralising capacity and the additional shock risk associated with poorly purified serum (6, 181).

Recently, success has been reported using novel immune-based therapies in animal models of TsV envenomation. In 2019, Zoccal et al. showed that the experimental peptide, EP80317 (a CD36 ligand), protected C57BL/6 mice against a lethal dose of TsV (97). Indeed, the therapeutic administration of EP80317 at 0.5 h and 2 h post-envenomation provided complete protection against a lethal dose of venom. Lymphocytes and neutrophils in the bronchoalveolar lavage fluid were significantly lower in the treatment group than venom alone. Accordingly, cAMP concentrations and proinflammatory cytokines (IL-1β, IL-6, TNF, and CCL3) were also considerably decreased (97).

Although promising, the estimated time and cost of developing a new drug and bringing it to market is 10 – 15 years and hundreds of millions of dollars (182). Conversely, drug repurposing circumvents the requirement for lengthy and expensive preclinical development. A recent *in vivo* study from the same group found that therapeutic administration with high dose dexamethasone (DEX) (5 mg/kg) improved TsV-induced cardiac dysfunction and reduced mortality after a fatal venom dose (170). The study showed that early treatment (15 min and 1 h post-inoculation) strongly suppressed PGE_2_ and IL-1β release in tissues, abrogating systemic ACh and IL1R-mediated/ACh-induced cardiac dysfunction (170).

In reviewing the effects of jellyfish venom on the immune system, Tibballs et al. highlighted similarities between the clinical features of Irukandji syndrome and scorpion envenomation (149). While the mechanisms underpinning the pathology of Irukandji syndrome have remained unresolved for decades, they may likewise involve both autonomic and inflammatory pathways. If so, immune-based therapies may also prove beneficial in severe box jellyfish envenomation and warrant further investigation.

### Irukandji Syndrome

While most jellyfish stings do not require medical attention, several species found in tropical waters constitute a public health threat (141). In Australia, the box jellyfish *Chironex fleckeri* and *Carukia barnesi* are of particular medical relevance. A high *C. fleckeri* venom dose can cause rapid and fatal cardiac arrest (183). In contrast, the smaller box jellyfish, *C. barnesi*, induces an extremely painful systemic pathology known as Irukandji syndrome (IS) (184–186).

**Figure 1:**
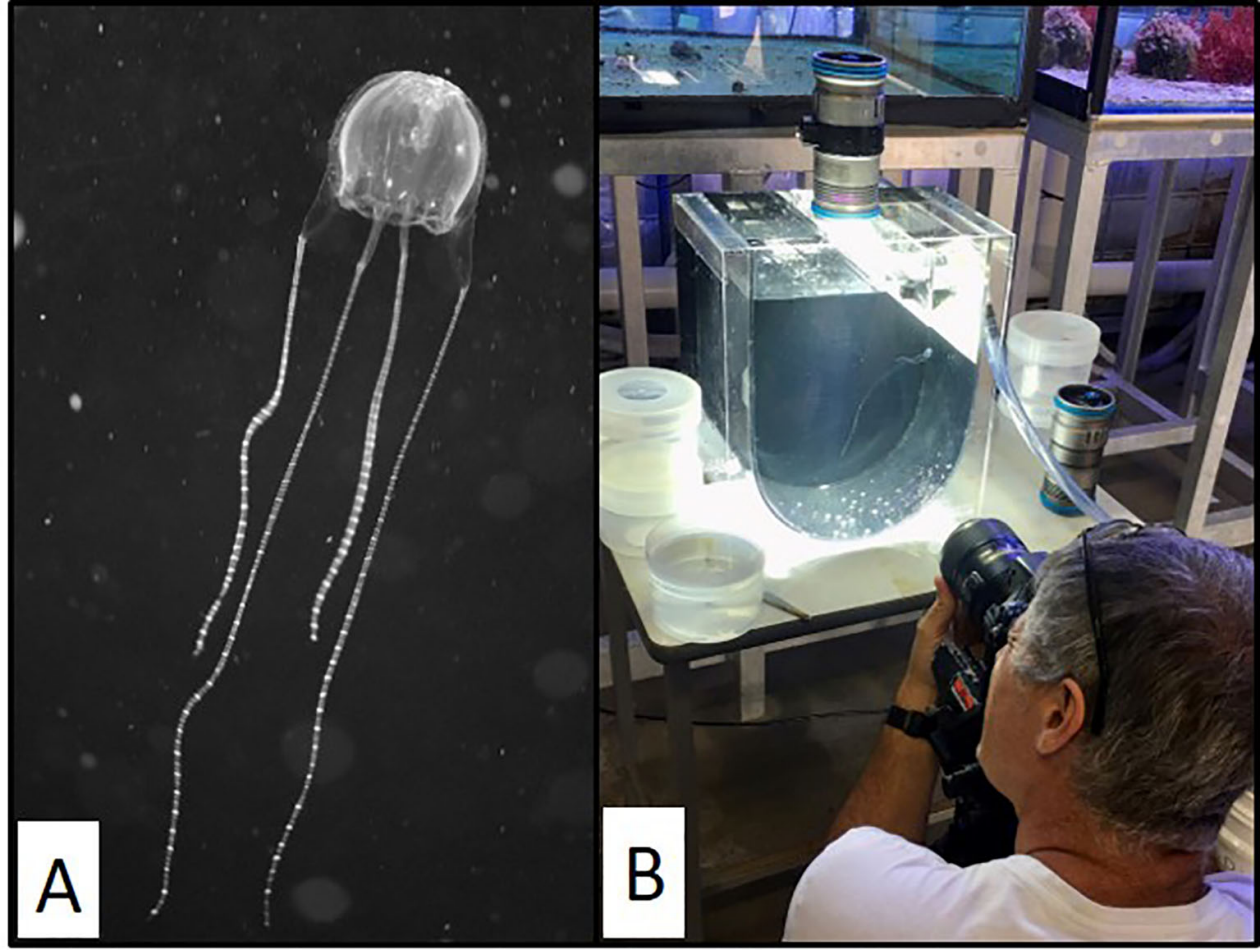
Image of Carukia barnesi jellyfish. Images showing **(A)** close up and **(B)** relative size of adult *C. barnesi* jellyfish. Prof Jamie Seymour pictured. Photos were taken by **(A)** Jamie Seymour (JCU, Cairns, Australia) and **(B)** Rachael Ryan (JCU, Cairns, Australia).

*C. barnesi* was the first confirmed causative agent of IS after its namesake, Dr Jack Barnes, famously subjected himself, his nine-year-old son, and a local lifeguard to intentional envenoming in 1961 (187). Yet, due in part to the elusive nature of this highly venomous jellyfish, research over the years has failed to unravel the mechanisms behind the distinctive syndromic illness (184, 188). *C. barnesi* are small and transparent, with the medusal bell measuring ~20 mm wide (**Figure 1**) (184, 189). As with other carybdeids, *C. barnesi* have a single tentacle per pedalium (184, 189). Both bell and tentacles are covered with nematocysts, comprising distinct venom composition (190).

**Figure 2:**
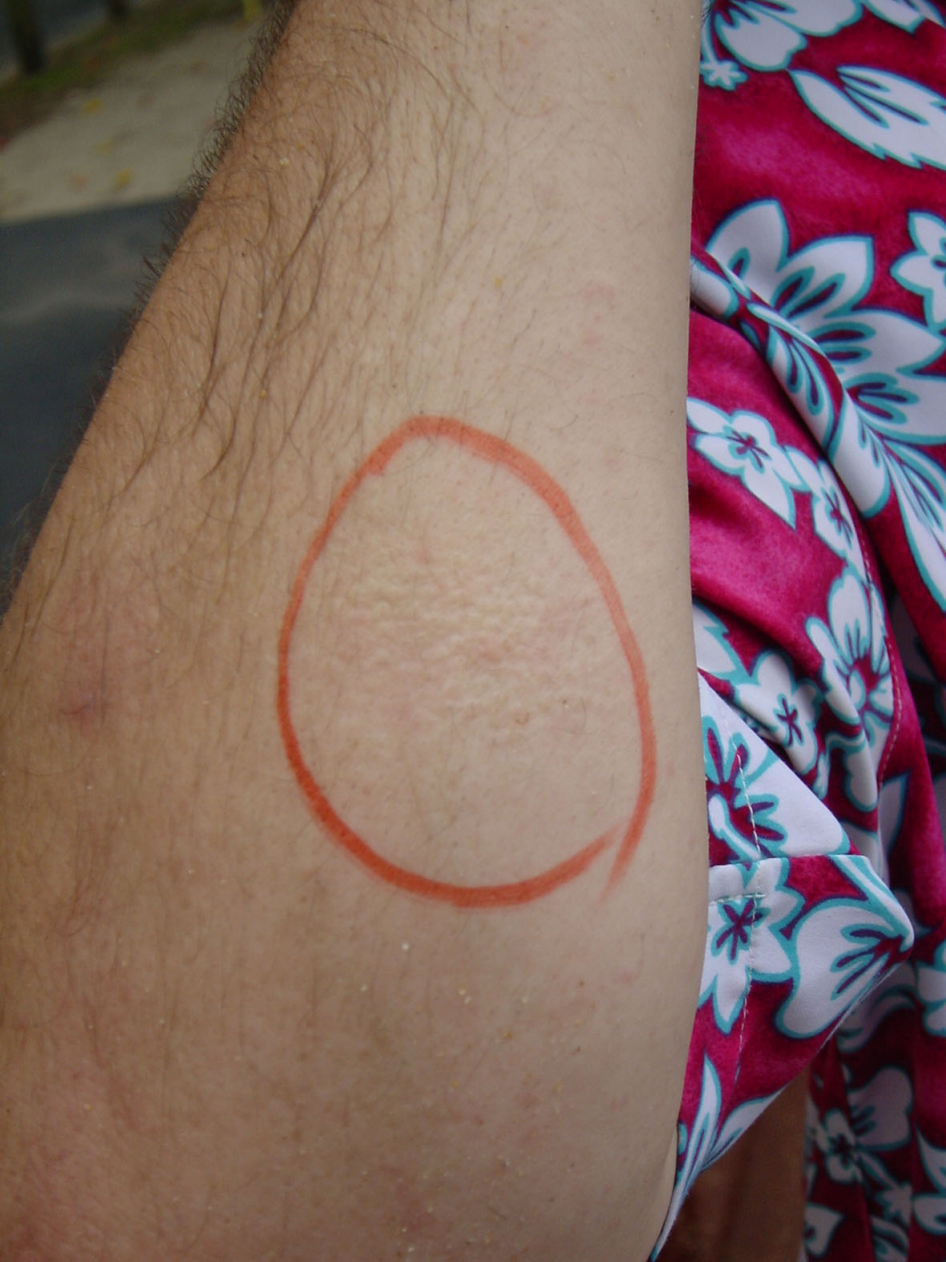
Local response to *C. barnesi* envenomation. Image showing a typical dermal reaction on the arm following a sting from a *C. barnesi* jellyfish. The red marker indicates the sting site. Photo by Jamie Seymour (JCU, Cairns, Australia).

A retrospective case study of 128 marine sting presentations to Cairns Base Hospital revealed a wide variation of symptom severity among individuals (186). Of the 39 patients with skin scrapings consistent with *C. barnesi* nematocysts, some experienced only minor symptoms, while in others, envenomation proved fatal (186). Typically, an IS presentation includes a mild local reaction followed by a characteristic incubation period of five to 60 min before the onset of systemic effects (**Figure 2**) (191, 192). Pain in the abdomen, chest, lower back, limbs, and joints, is severe, often intractable to opioids and accompanied by extreme distress and agitation (193, 194). In parallel, the manifestation of tachycardia, hypertension, diaphoresis, dyspnea, and in some instances, priapism may occur (193, 194). In severe cases, life-threatening complications, such as cardiomyopathy and cardiogenic shock, can arise (193). Due to the significant cardiac dysfunction associated with *C. barnesi* envenomation, cardiogenic pulmonary oedema may develop (195, 196). Tragically, venom-induced intracerebral hemorrhage resulted in the death of two individuals in 2002 (192, 197).

An “Irukandji” antivenom is unavailable, and, as *C. barnesi* is only one of several causative species, an antivenom is unlikely to be produced (198). Therefore, treatment of severe envenomation is heavily reliant upon opioid-based pain management and symptomatic supportive care, with a mean expected hospital stay of 1.6 days (186). The clinical manifestations of IS have been attributed to excessive catecholamine release, such as seen in pheochromocytoma, scorpionism, or funnel-web spider envenomation (149, 198, 199). As such, IS has been described as “a painful hypercatecholaminergic condition” (193). Accordingly, individuals at particular risk of fatal outcomes are those with pre-existing cardiovascular pathologies, potentially making the use of alpha/beta-adrenergic blocking agents prohibitive (200).

Supporting this hypothesis, adrenaline and noradrenaline have been transiently detected in the plasma of *C. barnesi* experimentally envenomed piglets (200). Peak catecholamine release was observed 10 min post intravenous (IV) venom administration, coinciding with the onset of systemic and pulmonary hypertension and tachycardia (200). Plasma catecholamines remained elevated in envenomed animals until 60 min but declined to non-significant levels within 2 h (200). Pretreatment with 1 µmol/L tetrodotoxin (TTX) attenuated tachycardia responses from rat and guinea-pig isolated right atria but did not significantly alter venom-induced contraction of rat mesenteric small arteries (200). These data suggest the presence of a presynaptic neuronal voltage-gated sodium channel agonist within the venom, as well as the presence of a TTX-insensitive vasoconstrictor (149, 198–200). While a physiological stress response towards IV administration of any toxin may similarly stimulate adrenaline and noradrenaline release, the authors reported the uniqueness of the reaction compared to other box jellyfish venom (200).

Research by Ramasamy et al. found pretreatment with prazosin (50 µg/kg) partially reduced tachycardia in *C. barnesi* envenomed rats, further supporting the role of endogenous catecholamines in the pathogenesis of IS (198). However, the residual pulse pressure observed in the study suggested the contribution of factors besides catecholamines (198). Furthermore, the result was not reproduced by Winkel et al. with 0.3 µM prazosin pretreatment, possibly due to dose- or time-dependent factors that were not clearly stated in either study (198, 200). Unfortunately, as both cardiovascular studies required the use of anesthetised animals, euthanised after 2 h, the critical evaluation of later time points was not possible (198). Yet, in sting victims, symptoms can remain for days, potentially requiring intensive care (186).

Regardless, in line with these findings, current clinical guidelines recommend magnesium sulphate (MgSO_4_) therapy to attenuate pain and suppress excessive catecholamine release in severe IS (201). Its success in doing so has generated divided opinions (202). The results of a randomised trial completed in 2012, and reviewed in 2017, were unable to confirm the ability of MgSO_4_ infusion to reduce opioid requirement (202, 203). Both studies reported varied success from the 39 patients, ultimately showing no significant benefit from MgSO_4_ therapy (202, 203).

Akin to scorpion envenomation, the symptoms of IS cannot be wholly attributed to sympathetic hyperstimulation (10). Also akin to scorpionism, generalised IS symptoms resemble those of CRS. Interestingly, MgSO_4_ potently suppresses MNC-mediated cytokine production following TLR stimulation (204). MgSO_4_ increases IκBα levels in MNCs, thereby decreasing NF-κB nuclear translocation and its activity (204). Accordingly, the ability of MgSO_4_ to inhibit pain in some sting patients could in part be due to a dampened immune response, although this theory has not been investigated.

Presently, neither catecholamines nor inflammatory mediators have been measured in *C. barnesi* sting patients. Recently, a study by Staedtke et al. proposed an intriguing link between “cytokine storm” and “catecholamine storm” in systemic inflammatory response syndrome (SIRS) and capillary leak syndrome, which may apply to venom-induced systemic inflammation (205, 206). This study showed that adrenaline contributes to the positive feed-forward cytokine dysregulation seen in CRS (206). Encouragingly, the blockade of α_1_-adrenergic receptors (also expressed on immune cells) or the inhibition of tyrosine hydroxylase (required for catecholamine biosynthesis) by prazosin or metyrosine hindered the self-amplifying proinflammatory loop *in vitro* and *in vivo* (206).

Specifically, CRS, induced by humanised CD19 CAR-T cells in mice engrafted with a leukemia cell line, caused excessive levels of adrenaline, noradrenaline, and myeloid-derived cytokines (IL-6, KC, MCP-1, and TNF) in the plasma of animals with high tumour burdens (206). Consequently, higher mortality was observed among these mice. In contrast, pretreatment with metyrosine or prazosin lowered circulating catecholamines and cytokines, improving survival (206). In mouse peritoneal MΦs, LPS-stimulation induced the release of catecholamines and proinflammatory cytokines, IL-6, KC, MCP-1, and TNF (206). Cytokine and catecholamine secretion was markedly enhanced in LPS/adrenaline co-cultures (206). Conversely, reduced MΦ catecholamine production significantly reduced IL-6, KC, MCP-1, and TNF levels (206). Although this model is unrelated to envenomation, it suggests that immune stimuli, such as venom-induced TLR activation, initiates a proinflammatory response that is enhanced by the presence of catecholamines. The dual stimulatory signals create a positive feed-forward loop, resulting in cytokine amplification. Collectively, these studies suggest a therapeutic potential of prazosin for severe IS, except where contraindicated, and warrant the investigation of plasma cytokines in sting patients.

However, while such research supports the therapeutic inhibition of catecholamines in envenomation, understanding their possible protective function has not been investigated. For example, it is known that catecholamine release promotes alveolar fluid clearance (174). In scorpionism, a rapid catecholamine surge following a dangerous sting is reasoned to increase alveolar fluid reabsorption, protecting the lungs from venom-induced flooding (174). Therefore, the resolution of the surge may decrease the ability of the lungs to clear venom-induced oedema, ultimately progressing toward fatal pulmonary oedema (174). Accordingly, animal models of IS should thoroughly scrutinise both the benefits and limitations of these mediators.

Finally, given the immune system’s propensity toward hyper-responsiveness and allergic reaction to jellyfish venom, the overlap in IS and CRS generalised symptoms, and the recently described link between CRS and catecholamine storm, immune involvement in the pathology is plausible. Nevertheless, to date, no *C. barnesi* immunology-based research has been published.

## The Immunosuppressive Potential of Venom-Derived Molecules

Despite the health burden of human envenomation, venom-immune interactions have been exploited in traditional medicine for centuries (207). More recently, research groups throughout the world have demonstrated the *in vitro* and *in vivo* efficacy of whole venom and venom-derived compounds in ameliorating a wide range of autoimmune symptoms (112, 208–211).

Presently, the most promising drug leads belong to the class of ion channel modulators. Ion channels, particularly calcium-activated and voltage-gated potassium channels, are attractive therapeutic targets for autoimmune diseases. Firstly, ion channels, such as the Shaker-related voltage-gated K_V_1.3 and ${\mathrm{CA}}_{-}^{2+}-$ dependent KCa3.1, regulate Ca^2+^ signalling in activated immune cells, allowing cell depolarisation and maintenance of membrane potential. Intracellular Ca^2+^ levels dictate T cell activation, proliferation, metabolism and cytokine production (212). Secondly, unique ion channel dimers are differentially expressed in various tissues, including immune cell subsets, permitting cell type and subset-specific blockade (213, 214). For example, activated effector memory T cells (T_EM_), B cells and MΦs, known mediators in the pathogenesis of various autoimmune diseases, preferentially upregulate K_V_1.3 (215–217). In contrast, naïve (T_n_) and central memory cells (T_CM_) express KCa3.1 ion channels, allowing for channel-specific inhibition (218). Finally, inhibition of Ca^2+^ influx *via* ion channel blockade allows targeted and reversible immune modulation, rather than complete T cell suppression, as induced by T cell Ca^2+^ modulating drugs, including calcineurin inhibitors and steroids.

Venom from snakes, spiders, scorpions, cone snails, and sea anemones comprise a diverse range of peptide and small molecule ion channel blockers that exhibit selectivity at picomolar concentrations (219). Blockade of lymphocyte ion channels using venom-derived compounds has therapeutic effects in animal models of rheumatoid arthritis (RA), asthma, multiple sclerosis (MS), delayed-type hypersensitivity, and allograft rejection (113, 208, 219–224). Notably, a selective peptide blocker, *Stichodactyla helianthus* toxin (ShK), from sea anemone venom, and anuroctoxin, a peptidyl toxin isolated from *Buthus sindicus* scorpion venom, have been shown to specifically target K_V_1.3 channels with high affinity, preventing Ca^2+^ influx and thereby inhibiting T_EM_ activation, proliferation and cytokine production (113, 115, 225–227).

Structural studies centred on the selectivity of peptide ion channel blockers have revealed that specificity is due to single amino acid effects rather than *en bloc* backbone structure (228). Thus, venom-derived peptides may act as promising drug scaffolds, notably because disulphide bonds encode robust biological stability (229). This has important implications for drug development, as synthetic manipulation may improve drug activity or remove toxicity from the natural peptide blueprint. For example, ShK(L5), a synthetic analog of ShK, contains an N-terminal L-phosphotyrosine extension and shows higher selectivity than the native peptide for K_V_1.3 channels over the neuronal ion channel K_V_1.1 (113).

Aside from ion channel blockade, venom-derived components have demonstrated potent *in vitro* and *in vivo* anti-inflammatory activity through the cholinergic anti-inflammatory pathway *via* an alpha7 nicotinic acetylcholine receptor antagonist (114). Venom-derived peptides, such as the α-neurotoxin from the Thailand cobra, are potent nicotinic receptor antagonists (218). In a rodent RA model, Cobratoxin-treatment reduced expression of the pro-inflammatory cytokines IL-1β, IL-2, and TNFα, resulting in decreased paw sensitivity and joint destruction (230).

Other neurotoxins, such as the principal toxin (NTX) from *Naja atra* venom (NNAV), have shown therapeutic effects in animal models of adjunctive arthritis, RA, Systemic Lupus Erythematosus (SLE), and nephropathy (209, 211). Additionally, NTX-treatment prolonged skin allograft survival in rats and inhibited cell-mediated immune responses in a dose-dependent manner through decreased Th1-type cytokines (IL-2 and IFN-γ). Although low NTX concentrations were cytotoxic, heat-treatment reduced NTX toxicity without reducing its immunosuppressive activity (211). In another study, orally administered NTX suppressed murine T cell proliferation, specifically Th17 and CD8^+^ T cell activity, increasing NK cell and B cell proliferation in a dose-dependent manner (209).

Venom from the honey bee has been used for centuries in traditional medicine to treat chronic inflammatory diseases due to its reported anti-inflammatory activity (207). Investigations into the mechanism of action of honey bee venom and its major components, melittin and phospholipase A2, have confirmed a protective effect in animal models of asthma and RA (207, 231). The polarisation of T cells towards a Th2 phenotype is associated with allergies and chronic inflammatory diseases (232). An essential driving factor in lineage determination is cytokine expression. It has been shown that melittin inhibits LPS-induced inflammation by binding to the C- terminus of the NF-κB p50 subunit, thus preventing translocation into the nucleus and transcription of pro-inflammatory cytokines, including TNF (233–235). Moreover, treatment with whole honey bee venom polarised T cells towards a Th1 phenotype by inducing T-bet and IFN-γ in CD4^+^ T cells (210). Conversely, PLA_2_, an enzyme found within the venom of multiple species, including the western honey bee, can hydrolyze membrane phospholipids and induce Th2 cytokine responses through the activation of ST2, a component of the IL-33 receptor on innate immune cells (236).

Other known venom immune modulators include tick salivary protein (Salp) 15 from *Ixodes scapularis* and spermine. Salp15 binds the CD4 co-receptor, MHC-II, inhibiting TCR ligation and T cell activation by misaligning CD4 with the TCR complex (237). Spermine, an acylpolyamine found in snake and spider venom, suppresses mitogen-induced activation and proliferation of PBMCs by inhibiting LAF-1 protein expression, involved in RNA remodelling (238).

Collectively, these studies highlight the potential of venom-derived molecules to modulate immune cells as unmodified venom-derived compounds or as scaffolds for drug development. Venom-derived compounds induce immune suppression using diverse modes of action. Thus, screening venom for its immunosuppressive and immune-activating potential may result in new immunomodulatory drugs and the discovery of new biological pathways.

## Conclusion

**Figure 3:**
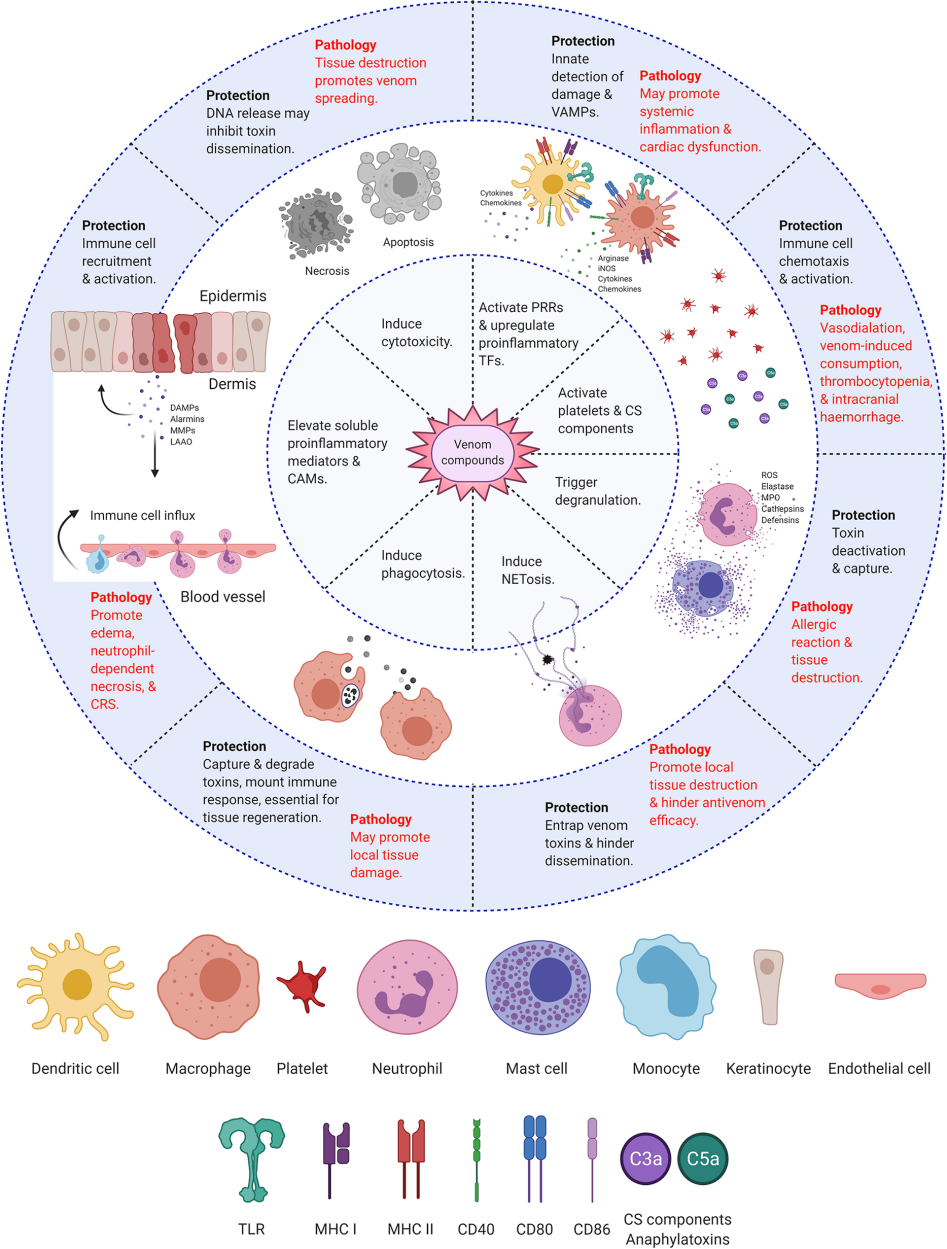
Immunological responses to envenomation. Diagram summarizing the protective and pathological responses of the host’s immune system towards venom compounds. Created with BioRender.com.

Conferring protection against venom’s potentially lethal action requires rapid immune recognition and response. Extensive research focuses on the degree to which immune responses themselves contribute to the severity of envenomation (**Figure 3**). However, there is disagreement regarding whether the body’s defensive reactions are helpful or harmful. Perhaps the most significant cause of division lies in the difficulty of distinguishing the actual venom-induced symptoms from immune-induced pathology. The classic inflammation markers (heat, pain, redness, swelling, and loss of function) are typical biological responses to envenomation across many species. Therefore, determining which symptoms are treatable using immunological approaches requires further research.

Nevertheless, venom’s ability to modulate immune activity has two therapeutic implications. Firstly, continued research could inform improved treatment strategies for fatal bites and stings. Secondly, as venom is a rich source of specific and potent biomodulators, exploring venom-immune interactions may lead to discovering novel pathways/receptors or the development of venom-derived immunomodulatory drugs.

## Author Contributions

Writing—original draft preparation, RR. Writing—review and editing, RR, JM, MI, JS, AL, and JL. Supervision, JM, MI, and JL. Funding acquisition, JM. All authors contributed to the article and approved the submitted version.

## Funding

This research received funding from the Australian National Health and Medical Council (NHMRC) (1031652 & 1131732). RR was supported by an Australian Government Research Training Program stipend. MI is supported by the TALENTO Program by the Regional Madrid Government (2018-T1/BIO-11262). JM (Career Development Fellowship APP1131732) is supported by the Australian National Health & Medical Research Council.

## Conflict of Interest

The authors declare that the research was conducted in the absence of any commercial or financial relationships that could be construed as a potential conflict of interest.

## References

[1] Jouiaei M Yanagihara AA Madio B Nevalainen TJ Alewood PF Fry BG. Ancient Venom Systems: A Review on Cnidaria Toxins. Toxins (2015) 7(6):2251–71. 10.3390/toxins7062251 PMC4488701 26094698

[2] Touchard A Aili SR Fox EGP Escoubas P Orivel J Nicholson GM. The Biochemical Toxin Arsenal From Ant Venoms. Toxins (2016) 8(1):30. 10.3390/toxins8010030 PMC4728552 26805882

[3] Chan YS Cheung RC Xia L Wong JH Ng TB Chan WY. Snake Venom Toxins: Toxicity and Medicinal Applications. Appl Microbiol Biotechnol (2016) 100(14):6165–81. 10.1007/s00253-016-7610-9 27245678

[4] King G Hardy M. Spider-Venom Peptides: Structure, Pharmacology, and Potential for Control of Insect Pests. Annu Rev Entomol (2012) 58:475–96. 10.1146/annurev-ento-120811-153650 23020618

[5] Gutiérrez JM Escalante T Rucavado A Herrera C Fox JW. A Comprehensive View of the Structural and Functional Alterations of Extracellular Matrix by Snake Venom Metalloproteinases (Svmps): Novel Perspectives on the Pathophysiology of Envenoming. Toxins (2016) 8(10):304. 10.3390/toxins8100304 PMC5086664 27782073

[6] Chippaux JP Goyffon M. Epidemiology of Scorpionism: A Global Appraisal. Acta Trop (2008) 107(2):71–9. 10.1016/j.actatropica.2008.05.021 18579104

[7] Helbling A Müller UR. Allergic Reactions to Stinging and Biting Insects, Fifth ed. Rich R Fleisher T Shearer W Schroeder H Frew A Weyand C, editors. London: Elsevier (2019). pp. 601–10.e1.

[8] Egawa G Kabashima K. Skin as a Peripheral Lymphoid Organ: Revisiting the Concept of Skin-Associated Lymphoid Tissues. J Invest Dermatol (2011) 131(11):2178–85. 10.1038/jid.2011.198 21734715

[9] Eyerich S Eyerich K Traidl-Hoffmann C Biedermann T. Cutaneous Barriers and Skin Immunity: Differentiating a Connected Network. Trends Immunol (2018) 39(4):315–27. 10.1016/j.it.2018.02.004 29551468

[10] Reis MB Zoccal KF Gardinassi LG Faccioli LH. Scorpion Envenomation and Inflammation: Beyond Neurotoxic Effects. Toxicon Off J Int Soc Toxinol (2019) 167:174–9. 10.1016/j.toxicon.2019.06.219 31228480

[11] Aristizábal B GÁ. Innate Immune System. Autoimmunity: From Bench to Bedside. Anaya JM SY Rojas-Villarraga A, editors. Bogota (Colombia: El Rosario University Press (2013). 29087650

[12] Mogensen TH. Pathogen Recognition and Inflammatory Signaling in Innate Immune Defenses. Clin Microbiol Rev (2009) 22(2):240–73. 10.1128/CMR.00046-08 PMC2668232 19366914

[13] Strbo N Yin N Stojadinovic O. Innate and Adaptive Immune Responses in Wound Epithelialization. Adv Wound Care (New Rochelle) (2014) 3(7):492–501. 10.1089/wound.2012.0435 25032069 PMC4086194

[14] Dunkelberger JR Song W-C. Complement and its Role in Innate and Adaptive Immune Responses. Cell Res (2010) 20(1):34–50. 10.1038/cr.2009.139 20010915

[15] Palm NW Medzhitov R. Role of the Inflammasome in Defense Against Venoms. Proc Natl Acad Sci U States America (2013) 110(5):1809–14. 10.1073/pnas.1221476110 PMC3562764 23297192

[16] Teixeira CF Zamuner SR Zuliani JP Fernandes CM Cruz-Hofling MA Fernandes I. Neutrophils do Not Contribute to Local Tissue Damage, But Play a Key Role in Skeletal Muscle Regeneration, in Mice Injected With Bothrops Asper Snake Venom. Muscle Nerve (2003) 28(4):449–59. 10.1002/mus.10453 14506717

[17] Xu H Timares L Elmets CA. Host Defenses in the Skin. In: Rich RR Fleisher TA Shearer WT Schroeder HW Frew AJ Weyand CM, editors. Clinical Immunology. Elsevier Saunders (2013). p. 228–38.

[18] Schlüter H Upjohn E Varigos G Kaur P. Burns and Skin Ulcers. In: Lanza R Atala A, editors. Essentials of Stem Cell Biology, 3rd ed. Boston: Academic Press (2014). p. 501–13.

[19] Albanesi C Madonna S Gisondi P Girolomoni G. The Interplay Between Keratinocytes and Immune Cells in the Pathogenesis of Psoriasis. Front Immunol (2018) 9:1549–. 10.3389/fimmu.2018.01549 PMC6043636 30034395

[20] Pivarcsi A Kemeny L Dobozy A. Innate Immune Functions of the Keratinocytes. A Rev Acta Microbiol Immunol Hungarica (2004) 51(3):303–10. 10.1556/AMicr.51.2004.3.8 15571070

[21] Lebre MC van der Aar AMG van Baarsen L van Capel TMM Schuitemaker JHN Kapsenberg ML. Human Keratinocytes Express Functional Toll-Like Receptor 3, 4, 5, and 9. J Invest Dermatol (2007) 127(2):331–41. 10.1038/sj.jid.5700530 17068485

[22] Costal-Oliveira F Stransky S Guerra-Duarte C Naves de Souza DL Vivas-Ruiz DE Yarlequé A. L-Amino Acid Oxidase From Bothrops Atrox Snake Venom Triggers Autophagy, Apoptosis and Necrosis in Normal Human Keratinocytes. Sci Rep (2019) 9(1):781–. 10.1038/s41598-018-37435-4 PMC6349910 30692577

[23] Stransky S Costal-Oliveira F Lopes-de-Souza L Guerra-Duarte C Chávez-Olórtegui C Braga VMM. In Vitro Assessment of Cytotoxic Activities of Lachesis Muta Muta Snake Venom. PloS neglected Trop Dis (2018) 12(4):e0006427. 10.1371/journal.pntd.0006427 PMC5919693 29659601

[24] Silva-de-Franca F Villas-Boas IM Serrano SMT Cogliati B Chudzinski SAA Lopes PH. Naja Annulifera Snake: New Insights Into the Venom Components and Pathogenesis of Envenomation. PloS Negl Trop Dis (2019) 13(1):e0007017. 10.1371/journal.pntd.0007017 30657756 PMC6338361

[25] Bhattacharjee P Mitra J Bhattacharyya D. L-Amino Acid Oxidase From Venoms. Dordrecht: Springer Netherlands (2017). p. 295–320.

[26] Paixão-Cavalcante D van den Berg CW de Freitas Fernandes-Pedrosa M Gonçalves de Andrade RM Tambourgi DV. Role of Matrix Metalloproteinases in HaCaT Keratinocytes Apoptosis Induced by Loxosceles Venom Sphingomyelinase D. J Invest Dermatol (2006) 126(1):61–8. 10.1038/sj.jid.5700049 16417218

[27] Telser A. Molecular Biology of the Cell. Shock. 18, 4th ed. New York: Garland Science (2002). p. 289. Available from: https://www.ncbi.nlm.nih.gov/books/NBK21054/.

[28] Al-Soudi A Kaaij MH Tas SW. Endothelial Cells: From Innocent Bystanders to Active Participants in Immune Responses. Autoimmun Rev (2017) 16(9):951–62. 10.1016/j.autrev.2017.07.008 28698091

[29] Khakpour S Wilhelmsen K Hellman J. Vascular Endothelial Cell Toll-like Receptor Pathways in Sepsis. Innate Immun (2015) 21(8):827–46. 10.1177/1753425915606525 26403174

[30] Pugin J Ulevitch RJ Tobias PS. Tumor Necrosis Factor-Alpha and Interleukin-1 Beta Mediate Human Endothelial Cell Activation in Blood At Low Endotoxin Concentrations. J Inflammation (1995) 45(1):49–55. 7583352

[31] Krishnaswamy G Kelley J Yerra L Smith JK Chi DS. Human Endothelium as a Source of Multifunctional Cytokines: Molecular Regulation and Possible Role in Human Disease. J Interferon Cytokine Res (1999) 19(2):91–104. 10.1089/107999099314234 10090394

[32] Teijaro John R Walsh Kevin B Cahalan S Fremgen Daniel M Roberts E Scott F. Endothelial Cells are Central Orchestrators of Cytokine Amplification During Influenza Virus Infection. Cell (2011) 146(6):980–91. 10.1016/j.cell.2011.08.015 PMC3176439 21925319

[33] Pober JS Merola J Liu R Manes TD. Antigen Presentation by Vascular Cells. Front Immunol (2017) 8:1907. 10.3389/fimmu.2017.01907 29312357 PMC5744398

[34] Joyce DE Nelson DR Grinnell BW. Leukocyte and Endothelial Cell Interactions in Sepsis: Relevance of the Protein C Pathway. Crit Care Med (2004) 32(5 Suppl):S280–6. 10.1097/01.CCM.0000128037.72072.22 15118531

[35] Nourshargh S Alon R. Leukocyte Migration Into Inflamed Tissues. Immunity (2014) 41(5):694–707. 10.1016/j.immuni.2014.10.008 25517612

[36] Krieglstein CF Granger DN. Adhesion Molecules and Their Role in Vascular Disease. Am J Hypertens (2001) 14(6 Pt 2):44s–54s. 10.1016/S0895-7061(01)02069-6 11411765

[37] Danese S Dejana E Fiocchi C. Immune Regulation by Microvascular Endothelial Cells: Directing Innate and Adaptive Immunity, Coagulation, and Inflammation. J Immunol (Baltimore Md 1950) (2007) 178(10):6017–22. 10.4049/jimmunol.178.10.6017 17475823

[38] Borkow G Lomonte B Gutierrez JM Ovadia M. Effect of Various Viperidae and Crotalidae Snake Venoms on Endothelial Cells In Vitro. Toxicon Off J Int Soc Toxinol (1994) 32(12):1689–95. 10.1016/0041-0101(94)90330-1 7725339

[39] Nowatzki J de Sene RV Paludo KS Veiga SS Oliver C Jamur MC. Brown Spider Venom Toxins Interact With Cell Surface and are Endocytosed by Rabbit Endothelial Cells. Toxicon Off J Int Soc Toxinol (2010) 56(4):535–43. 10.1016/j.toxicon.2010.03.027 20573594

[40] Chung C-H Wu W-B Huang T-F. Aggretin, a Snake Venom–Derived Endothelial Integrin α2β1 Agonist, Induces Angiogenesis Via Expression of Vascular Endothelial Growth Factor. Blood (2004) 103(6):2105–13. 10.1182/blood-2003-07-2483 14630793

[41] Paludo KS Gremski LH Veiga SS Chaim OM Gremski W de Freitas Buchi D. The Effect of Brown Spider Venom on Endothelial Cell Morphology and Adhesive Structures. Toxicon Off J Int Soc Toxinol (2006) 47(8):844–53. 10.1016/j.toxicon.2006.02.006 16737725

[42] Delafontaine M Villas-Boas IM Mathieu L Josset P Blomet J Tambourgi DV. Enzymatic and Pro-Inflammatory Activities of Bothrops Lanceolatus Venom: Relevance for Envenomation. Toxins (2017) 9(8):244. 10.3390/toxins9080244 PMC5577578 28783135

[43] Patel KD Modur V Zimmerman GA Prescott SM McIntyre TM. The Necrotic Venom of the Brown Recluse Spider Induces Dysregulated Endothelial Cell-Dependent Neutrophil Activation. Differential Induction of GM-CSF, Il-8, and E-selectin Expression. J Clin Invest (1994) 94(2):631–42. 10.1172/JCI117379 PMC296140 7518841

[44] Chiu S Bharat A. Role of Monocytes and Macrophages in Regulating Immune Response Following Lung Transplantation. Curr Opin Organ Transplant (2016) 21(3):239–45. 10.1097/MOT.0000000000000313 PMC4858348 26977996

[45] Randolph GJ Jakubzick C Qu C. Antigen Presentation by Monocytes and Monocyte-Derived Cells. Curr Opin Immunol (2008) 20(1):52–60. 10.1016/j.coi.2007.10.010 18160272 PMC2408874

[46] Franken L Schiwon M Kurts C. Macrophages: Sentinels and Regulators of the Immune System. Cell Microbiol (2016) 18(4):475–87. 10.1111/cmi.12580 26880038

[47] Ma WT Gao F Gu K Chen DK. The Role of Monocytes and Macrophages in Autoimmune Diseases: A Comprehensive Review. Front Immunol (2019) 10:1140. 10.3389/fimmu.2019.01140 31178867 PMC6543461

[48] Sampaio SC Sousa-e-Silva MC Borelli P Curi R Cury Y. Crotalus Durissus Terrificus Snake Venom Regulates Macrophage Metabolism and Function. J Leukocyte Biol (2001) 70(4):551–8. 11590191

[49] Setubal SS Pontes AS Furtado JL Kayano AM Stábeli RG Zuliani JP. Effect of Bothrops Alternatus Snake Venom on Macrophage Phagocytosis and Superoxide Production: Participation of Protein Kinase C. J Venomous Anim Toxins Including Trop Dis (2011) 17(4):430–41. 10.1590/S1678-91992011000400010

[50] Saadi S Assarehzadegan MA Pipelzadeh MH Hadaddezfuli R. Induction of IL-12 From Human Monocytes After Stimulation With Androctonus Crassicauda Scorpion Venom. Toxicon Off J Int Soc Toxinol (2015) 106:117–21. 10.1016/j.toxicon.2015.09.029 26415903

[51] Pires WL Kayano AM Castro OB Paloschi MV Lopes JA Boeno CN. Lectin Isolated From Bothrops Jararacussu Venom Induces IL-10 Release by TCD4+ Cells and TNF-α Release by Monocytes and Natural Killer Cells. J leukocyte Biol (2019) 106(3):595–605. 10.1002/JLB.MA1118-463R 31087703

[52] Rojas JM Aran-Sekul T Cortes E Jaldín R Ordenes K Orrego PR. Phospholipase D From Loxosceles Laeta Spider Venom Induces IL-6, Il-8, Cxcl1/Gro-α, and CCL2/MCP-1 Production in Human Skin Fibroblasts and Stimulates Monocytes Migration. Toxins (2017) 9(4):125. 10.3390/toxins9040125 PMC5408199 28379166

[53] Zoccal KF Ferreira GZ Prado MKB Gardinassi LG Sampaio SV Faccioli LH. LTB4 and PGE2 Modulate the Release of MIP-1α and IL-1β by Cells Stimulated With Bothrops Snake Venoms. Toxicon Off J Int Soc Toxinol (2018) 150:289–96. 10.1016/j.toxicon.2018.06.066 29894720

[54] Khemili D Valenzuela C Laraba-Djebari F Hammoudi-Triki D. Differential Effect of Androctonus Australis Hector Venom Components on Macrophage KV Channels: Electrophysiological Characterization. Eur Biophys J (2019) 48(1):1–13. 10.1007/s00249-018-1323-1 30006779

[55] Hsu C-C Chuang W-J Chung C-H Chang C-H Peng H-C Huang T-F. Snake Venom Disintegrin Inhibits the Activation of Toll-Like Receptors and Alleviates Sepsis Through Integrin alphaVbeta3 Blockade. Sci Rep (Nature Publisher Group) (2016) 6:23387. 10.1038/srep23387 PMC4796821 26987407

[56] Zoccal KF Bitencourt C Paula-Silva FWG CA S de Castro Figueiredo Bordon K Arantes EC. Tlr2, TLR4 and CD14 Recognize Venom-Associated Molecular Patterns From Tityus Serrulatus to Induce Macrophage-Derived Inflammatory Mediators. PloS One (2014) 9(2):e88174–e. 10.1371/journal.pone.0088174 PMC3917877 24516606

[57] Veda P. Why are Neutrophils Polymorphonuclear? Eur J Inflammation (2011) 9(2):85–93. 10.1177/1721727X1100900201

[58] Kruger P Saffarzadeh M Weber ANR Rieber N Radsak M von Bernuth H. Neutrophils: Between Host Defence, Immune Modulation, and Tissue Injury. PloS Pathog (2015) 11(3):e1004651. 10.1371/journal.ppat.1004651 25764063 PMC4357453

[59] Summers C Rankin SM Condliffe AM Singh N Peters AM Chilvers ER. Neutrophil Kinetics in Health and Disease. Trends Immunol (2010) 31(8):318–24. 10.1016/j.it.2010.05.006 PMC2930213 20620114

[60] Pillay J den Braber I Vrisekoop N Kwast LM de Boer RJ Borghans JA. In Vivo Labeling With 2H2O Reveals a Human Neutrophil Lifespan of 5. 4 days. Blood (2010) 116(4):625–7. 10.1182/blood-2010-01-259028 20410504

[61] Tecchio C Micheletti A Cassatella MA. Neutrophil-Derived Cytokines: Facts Beyond Expression. Front Immunol (2014) 5:508–. 10.3389/fimmu.2014.00508 PMC4204637 25374568

[62] Rorvig S Ostergaard O Heegaard NH Borregaard N. Proteome Profiling of Human Neutrophil Granule Subsets, Secretory Vesicles, and Cell Membrane: Correlation With Transcriptome Profiling of Neutrophil Precursors. J leukocyte Biol (2013) 94(4):711–21. 10.1189/jlb.1212619 23650620

[63] Papayannopoulos V. Neutrophil Extracellular Traps in Immunity and Disease. Nat Rev Immunol (2018) 18(2):134–47. 10.1038/nri.2017.105 28990587

[64] Kaplan MJ Radic M. Neutrophil Extracellular Traps: Double-edged Swords of Innate Immunity. J Immunol (Baltimore Md 1950) (2012) 189(6):2689–95. 10.4049/jimmunol.1201719 PMC3439169 22956760

[65] Katkar GD Sundaram MS NaveenKumar SK Swethakumar B Sharma RD Paul M. Netosis and Lack of DNase Activity are Key Factors in Echis Carinatus Venom-Induced Tissue Destruction. Nat Commun (2016) 7:11361. 10.1038/ncomms11361 27093631 PMC4838891

[66] Stackowicz J Balbino B Todorova B Godon O Iannascoli B Jönsson F. Evidence That Neutrophils do Not Promote Echis Carinatus Venom-Induced Tissue Destruction. Nat Commun (2018) 9(1):2304. 10.1038/s41467-018-04688-6 29899337 PMC5998045

[67] Williams DJ Faiz MA Abela-Ridder B Ainsworth S Bulfone TC Nickerson AD. Strategy for a Globally Coordinated Response to a Priority Neglected Tropical Disease: Snakebite Envenoming. PloS Negl Trop Dis (2019) 13(2):e0007059. 10.1371/journal.pntd.0007059 30789906 PMC6383867

[68] Krystel-Whittemore M Dileepan KN Wood JG. Mast Cell: A Multi-Functional Master Cell. Front Immunol (2016) 6:620–. 10.3389/fimmu.2015.00620 PMC4701915 26779180

[69] Galli SJ Starkl P Marichal T Tsai M. Mast Cells and IgE in Defense Against Venoms: Possible “Good Side” of Allergy? Allergology Int Off J Japanese Soc Allergology (2016) 65(1):3–15. 10.1016/j.alit.2015.09.002 26666482

[70] Rivera J Gilfillan AM. Molecular Regulation of Mast Cell Activation. J Allergy Clin Immunol (2006) 117(6):1214–25. 10.1016/j.jaci.2006.04.015 16750977

[71] Moon TC Befus AD Kulka M. Mast Cell Mediators: Their Differential Release and the Secretory Pathways Involved. Front Immunol (2014) 5:569–. 10.3389/fimmu.2014.00569 PMC4231949 25452755

[72] Gilfillan AM Tkaczyk C. Integrated Signalling Pathways for Mast-Cell Activation. Nat Rev Immunol (2006) 6(3):218–30. 10.1038/nri1782 16470226

[73] Metcalfe DD Peavy RD Gilfillan AM. Mechanisms of Mast Cell Signaling in Anaphylaxis. J Allergy Clin Immunol (2009) 124(4):639–48. 10.1016/j.jaci.2009.08.035 PMC2788154 19815110

[74] Galli SJ Tsai M. Ige and Mast Cells in Allergic Disease. Nat Med (2012) 18(5):693–704. 10.1038/nm.2755 22561833 PMC3597223

[75] Galli SJ Starkl P Marichal T Tsai M. Mast Cells and IgE can Enhance Survival During Innate and Acquired Host Responses to Venoms. Trans Am Clin Climatological Assoc (2017) 128:193–221. PMC5525434 28790503

[76] Pejler G Rönnberg E Waern I Wernersson S. Mast Cell Proteases: Multifaceted Regulators of Inflammatory Disease. Blood (2010) 115(24):4981–90. 10.1182/blood-2010-01-257287 20233968

[77] Fehervari Z. Mast Cells Crack Down on Venom. Nat Immunol (2011) 12(12):1141–. 10.1038/ni.2177

[78] Akdis M Aab A Altunbulakli C Azkur K Costa RA Crameri R. Interleukins (From IL-1 to IL-38), Interferons, Transforming Growth Factor Beta, and TNF-alpha: Receptors, Functions, and Roles in Diseases. J Allergy Clin Immunol (2016) 138(4):984–1010. 10.1016/j.jaci.2016.06.033 27577879

[79] Miller MC Mayo KH. Chemokines From a Structural Perspective. Int J Mol Sci (2017) 18(10):2088. 10.3390/ijms18102088 PMC5666770 28974038

[80] Oppenheim JJ Matsushima K Yoshimura T Leonard EJ. The Activities of Cytokines are Pleiotropic and Interdependent. Immunol Lett (1987) 16(3-4):179–83. 10.1016/0165-2478(87)90145-3 3327809

[81] Ozaki K Leonard WJ. Cytokine and Cytokine Receptor Pleiotropy and Redundancy. J Biol Chem (2002) 277(33):29355–8. 10.1074/jbc.R200003200 12072446

[82] Aikawa N. Cytokine Storm in the Pathogenesis of Multiple Organ Dysfunction Syndrome Associated With Surgical Insults. Nihon Geka Gakkai zasshi (1996) 97(9):771–7. 8940690

[83] Bown MJ Nicholson ML Bell PR Sayers RD. Cytokines and Inflammatory Pathways in the Pathogenesis of Multiple Organ Failure Following Abdominal Aortic Aneurysm Repair. Eur J Vasc endovascular Surg Off J Eur Soc Vasc Surg (2001) 22(6):485–95. 10.1053/ejvs.2001.1522 11735196

[84] De Gaudio AR Romagnoli S. Sepsis and Septic Shock, Eighth ed. China: Elsevier Health Sciences (2019). pp. 836–48.

[85] Lipnik-Stangelj M. Mediators of Inflammation as Targets for Chronic Pain Treatment. Mediators Inflammation (2013) 2013:783235–. 10.1155/2013/783235 PMC3848385 24347834

[86] Zhang J-M An J. Cytokines, Inflammation, and Pain. Int Anesthesiol Clin (2007) 45(2):27–37. 10.1097/AIA.0b013e318034194e 17426506 PMC2785020

[87] Chen L Deng H Cui H Fang J Zuo Z Deng J. Inflammatory Responses and Inflammation-Associated Diseases in Organs. Oncotarget (2017) 9(6):7204–18. 10.18632/oncotarget.23208 PMC5805548 29467962

[88] Robinson SM Rasch S Beer S Valantiene I Mickevicius A Schlaipfer E. Systemic Inflammation Contributes to Impairment of Quality of Life in Chronic Pancreatitis. Sci Rep (2019) 9(1):7318. 10.1038/s41598-019-43846-8 31086257 PMC6513859

[89] Henden AS Hill GR. Cytokines in Graft-Versus-Host Disease. J Immunol (2015) 194(10):4604. 10.4049/jimmunol.1500117 25934923

[90] Tisoncik JR Korth MJ Simmons CP Farrar J Martin TR Katze MG. Into the Eye of the Cytokine Storm. Microbiol Mol Biol Rev (2012) 76(1):16–32. 10.1128/MMBR.05015-11 22390970 PMC3294426

[91] White M. Mediators of Inflammation and the Inflammatory Process. J Allergy Clin Immunol (1999) 103(3):S378–S81. 10.1016/S0091-6749(99)70215-0 10069896

[92] Stone SF Isbister GK Shahmy S Mohamed F Abeysinghe C Karunathilake H. Immune Response to Snake Envenoming and Treatment With Antivenom; Complement Activation, Cytokine Production and Mast Cell Degranulation. PloS Neglected Trop Dis (2013) 7(7):e2326. 10.1371/journal.pntd.0002326 PMC3723557 23936562

[93] Ibiapina HNS Costa AG Sachett JAG Silva IM Tarrago AM Neves JCF. An Immunological Stairway to Severe Tissue Complication Assembly in Bothrops Atrox Snakebites. Front Immunol (2019) 10:1882. 10.3389/fimmu.2019.01882 31474982 PMC6705225

[94] Markland FS. Snake Venoms and the Hemostatic System. Toxicon Off J Int Soc Toxinol (1998) 36(12):1749–800. 10.1016/S0041-0101(98)00126-3 9839663

[95] Luchini LSG Pidde G Squaiella-Baptistão CC Tambourgi DV. Complement System Inhibition Modulates the Pro-Inflammatory Effects of a Snake Venom Metalloproteinase. Front Immunol (2019) 10:1137–. 10.3389/fimmu.2019.01539 PMC6558526 31231362

[96] Rider P Carmi Y Cohen I. Biologics for Targeting Inflammatory Cytokines, Clinical Uses, and Limitations. Int J Cell Biol (2016) 2016:9259646–. 10.1155/2016/9259646 PMC5204077 28083070

[97] Zoccal KF Gardinassi LG Bordon KCF Arantes EC Marleau S Ong H. EP80317 Restrains Inflammation and Mortality Caused by Scorpion Envenomation in Mice. Front Pharmacol (2019) 10:171. 10.3389/fphar.2019.00171 30886580 PMC6409428

[98] Dalla-Favera R Klein U. Germinal Centres: Role in B-cell Physiology and Malignancy. Nat Rev Immunol (2008) 8(1):22–33. 10.1038/nri2217 18097447

[99] Koretzky GA. Multiple Roles of CD4 and CD8 in T Cell Activation. J Immunol (2010) 185(5):2643–4. 10.4049/jimmunol.1090076 20724729

[100] Parker DC. T Cell-Dependent B Cell Activation. Annu Rev Immunol (1993) 11(1):331–60. 10.1146/annurev.iy.11.040193.001555 8476565

[101] Harwood NE Batista FD. Early Events in B Cell Activation. Annu Rev Immunol (2010) 28(1):185–210. 10.1146/annurev-immunol-030409-101216 20192804

[102] Restano-Cassulini R Garcia W Paniagua-Solís J Possani L. Annex 5: Guidelines for the Production, Control and Regulation of Snake Antivenom Immunoglobulins: Replacement of Annex 2 of WHO Technical Report Series, No. 964. World Health Organization (2016). Report No.: 0512-3054 Contract No.: Report.

[103] Fox S Rathuwithana AC Kasturiratne A Lalloo DG de Silva HJ. Underestimation of Snakebite Mortality by Hospital Statistics in the Monaragala District of Sri Lanka. Trans R Soc Trop Med Hygiene (2006) 100(7):693–5. 10.1016/j.trstmh.2005.09.003 16289649

[104] Gilliam LL Carmichael RC Holbrook TC Taylor JM Ownby CL McFarlane D. Antibody Responses to Natural Rattlesnake Envenomation and a Rattlesnake Toxoid Vaccine in Horses. Clin Vaccine Immunol (2013) 20(5):732–7. 10.1128/CVI.00004-13 PMC3647753 23515015

[105] Casadevall A. Passive Antibody Administration (Immediate Immunity) as a Specific Defense Against Biological Weapons. Emerg Infect Dis (2002) 8(8):833–41. 10.3201/eid0808.010516 PMC3369592 12141970

[106] Restano-Cassulini R Garcia W Paniagua-Solis JF Possani LD. Antivenom Evaluation by Electrophysiological Analysis. Toxins (2017) 9(3):74. 10.3390/toxins9030074 PMC5371829 28241514

[107] Elgueta R Benson MJ de Vries VC Wasiuk A Guo Y Noelle RJ. Molecular Mechanism and Function of CD40/CD40L Engagement in the Immune System. Immunol Rev (2009) 229(1):152–72. 10.1111/j.1600-065X.2009.00782.x PMC3826168 19426221

[108] Magee CN Boenisch O Najafian N. The Role of Costimulatory Molecules in Directing the Functional Differentiation of Alloreactive T Helper Cells. Am J Transplant (2012) 12(10):2588–600. 10.1111/j.1600-6143.2012.04180.x PMC3459149 22759274

[109] Vazquez MI Catalan-Dibene J Zlotnik A. B Cells Responses and Cytokine Production are Regulated by Their Immune Microenvironment. Cytokine (2015) 74(2):318–26. 10.1016/j.cyto.2015.02.007 PMC4475485 25742773

[110] Shlomchik MJ Weisel F. Germinal Center Selection and the Development of Memory B and Plasma Cells. Immunol Rev (2012) 247(1):52–63. 10.1111/j.1600-065X.2012.01124.x 22500831

[111] Khan U Ghazanfar H. T Lymphocytes and Autoimmunity. Int Rev Cell Mol Biol (2018) 341:125–68. 10.1016/bs.ircmb.2018.05.008 30262031

[112] Rangel-Santos A Lima C Lopes-Ferreira M Cardoso DF. Immunosuppresive Role of Principal Toxin (Crotoxin) of Crotalus Durissus Terrificus Venom. Toxicon Off J Int Soc Toxinol (2004) 44(6):609–16. 10.1016/j.toxicon.2004.07.004 15501286

[113] Beeton C Pennington MW Wulff H Singh S Nugent D Crossley G. Targeting Effector Memory T Cells With a Selective Peptide Inhibitor of Kv1.3 Channels for Therapy of Autoimmune Diseases. Mol Pharmacol (2005) 67(4):1369–81. 10.1124/mol.104.008193 PMC4275123 15665253

[114] Bencherif M Lippiello PM Lucas R Marrero MB. Alpha7 Nicotinic Receptors as Novel Therapeutic Targets for Inflammation-Based Diseases. Cell Mol Life Sci (2011) 68(6):931–49. 10.1007/s00018-010-0525-1 PMC3678737 20953658

[115] Bagdány M Batista CVF Valdez-Cruz NA Somodi S Rodriguez De La Vega RC Licea AF. Anuroctoxin, a New Scorpion Toxin of the α-KTx 6 Subfamily, is Highly Selective for Kv1.3 Over IKCa1 Ion Channels of Human T Lymphocytes. Mol Pharmacol (2005) 67(4):1034–44. 10.1124/mol.104.007187 15615696

[116] Waheed H Moin SF Choudhary MI. Snake Venom: From Deadly Toxins to Life-Saving Therapeutics. Curr Med Chem (2017) 24(17):1874–91. 10.2174/0929867324666170605091546 28578650

[117] Pennington MW Czerwinski A Norton RS. Peptide Therapeutics From Venom: Current Status and Potential. Bioorg Med Chem (2018) 26(10):2738–58. 10.1016/j.bmc.2017.09.029 28988749

[118] Bogin O. Venom Peptides and Their Mimetics as Potential Drugs. Modulator (2005) 19:14–20.

[119] Pineda SS Undheim EA Rupasinghe DB Ikonomopoulou MP King GF. Spider Venomics: Implications for Drug Discovery. Fut Med Chem (2014) 6(15):1699–714. 10.4155/fmc.14.103 25406008

[120] King GF. Venoms as a Platform for Human Drugs: Translating Toxins Into Therapeutics. Expert Opin Biol Ther (2011) 11(11):1469–84. 10.1517/14712598.2011.621940 21939428

[121] Ryan RYM Lutzky VP Herzig V Smallwood TB Potriquet J Wong Y. Venom of the Red-Bellied Black Snake Pseudechis Porphyriacus Shows Immunosuppressive Potential. Toxins (2020) 12(11):674. 10.3390/toxins12110674 PMC7693913 33114591

[122] Welton RE Williams DJ Liew D. Injury Trends From Envenoming in Australia, 2000–2013. Internal Med J (2017) 47(2):170–6. 10.1111/imj.13297 27749012

[123] Mullins RJ Wainstein BK Barnes EH Liew WK Campbell DE. Increases in Anaphylaxis Fatalities in Australia From 1997 to 2013. Clin Exp Allergy (2016) 46(8):1099–110. 10.1111/cea.12748 27144664

[124] Pesek RD Lockey RF. Management of Insect Sting Hypersensitivity: An Update. Allergy Asthma Immunol Res (2013) 5(3):129–37. 10.4168/aair.2013.5.3.129 PMC3636446 23638310

[125] Bilò MB. Anaphylaxis Caused by Hymenoptera Stings: From Epidemiology to Treatment. Allergy (2011) 66(95):35–7. 10.1111/j.1398-9995.2011.02630.x 21668850

[126] Curotto de Lafaille MA Lafaille JJ Graça L. Mechanisms of Tolerance and Allergic Sensitization in the Airways and the Lungs. Curr Opin Immunol (2010) 22(5):616–22. 10.1016/j.coi.2010.08.014 PMC3900231 20884192

[127] Ollert M Blank S. Anaphylaxis to Insect Venom Allergens: Role of Molecular Diagnostics. Curr Allergy Asthma Rep (2015) 15(5):26–. 10.1007/s11882-015-0527-z PMC4490169 26139335

[128] Janeway C National Library of M. Immunobiology: The Immune System in Health and Disease. Fifth ed. New York: Garland Pub (2001).

[129] van Ree R Hummelshøj L Plantinga M Poulsen LK Swindle E. Allergic Sensitization: Host-immune Factors. Clin Trans Allergy (2014) 4(1):12. 10.1186/2045-7022-4-12 PMC3989850 24735802

[130] Ellenbogen Y Jiménez-Saiz R Spill P Chu DK Waserman S Jordana M. The Initiation of Th2 Immunity Towards Food Allergens. Int J Mol Sci (2018) 19(5):1447. 10.3390/ijms19051447 PMC5983584 29757238

[131] Thangam EB Jemima EA Singh H Baig MS Khan M Mathias CB. The Role of Histamine and Histamine Receptors in Mast Cell-Mediated Allergy and Inflammation: The Hunt for New Therapeutic Targets. Front Immunol (2018) 9(1873). 10.3389/fimmu.2018.01873 PMC6099187 30150993

[132] Stitt JMD Katial RMD. Venom Allergy. J Allergy Clin Immunol: In Pract (2015) 4(1):184–5. 10.1016/j.jaip.2015.09.016 26772932

[133] Lieberman P. Biphasic Anaphylactic Reactions. Ann allergy Asthma Immunol Off Publ Am Coll Allergy Asthma Immunol (2005) 95(3):217–26; quiz 26, 58. 10.1016/S1081-1206(10)61217-3 16200811

[134] Liu M Yokomizo T. The Role of Leukotrienes in Allergic Diseases. Allergology Int (2015) 64(1):17–26. 10.1016/j.alit.2014.09.001 25572555

[135] Niedoszytko M Bonadonna P Oude Elberink JN Golden DB. Epidemiology, Diagnosis, and Treatment of Hymenoptera Venom Allergy in Mastocytosis Patients. Immunol Allergy Clinics North America (2014) 34(2):365–81. 10.1016/j.iac.2014.02.004 24745680

[136] Philipp AMD Ferdman RMMDM Tam JSMD. Evaluation of Venom Allergy. Ann Allergy Asthma Immunol (2016) 117(4):344–7. 10.1016/j.anai.2016.08.012 27742083

[137] Lieberman P. The Use of Antihistamines in the Prevention and Treatment of Anaphylaxis and Anaphylactoid Reactions. J Allergy Clin Immunol (1990) 86(4 Pt 2):684–6. 10.1016/S0091-6749(05)80241-6 1977785

[138] Remigante A Costa R Morabito R La Spada G Marino A Dossena S. Impact of Scyphozoan Venoms on Human Health and Current First Aid Options for Stings. Toxins (2018) 10(4):133. 10.3390/toxins10040133 PMC5923299 29570625

[139] Schiener M Graessel A Ollert M Schmidt-Weber CB Blank S. Allergen-Specific Immunotherapy of Hymenoptera Venom Allergy - Also a Matter of Diagnosis. Hum Vaccin Immunother (2017) 13(10):2467–81. 10.1080/21645515.2017.1334745 PMC5647953 28604163

[140] Mattila P Joenväärä S Renkonen J Toppila-Salmi S Renkonen R. Allergy as an Epithelial Barrier Disease. Clin Trans Allergy (2011) 1(1):1–8. 10.1186/2045-7022-1-5 PMC3294629 22410284

[141] Cegolon L Heymann WC Lange JH Mastrangelo G. Jellyfish Stings and Their Management: A Review. Mar Drugs (2013) 11(2):523–50. 10.3390/md11020523 PMC3640396 23434796

[142] Montgomery L Seys J Mees J. To Pee, or Not to Pee: A Review on Envenomation and Treatment in European Jellyfish Species. Mar Drugs (2016) 14(7):127. 10.3390/md14070127 PMC4962017 27399728

[143] Glatstein M Adir D Galil B Scolnik D Rimon A Pivko-Levy D. Pediatric Jellyfish Envenomation in the Mediterranean Sea. Eur J Emergency Med Off J Eur Soc Emergency Med (2018) 25(6):434–9. 10.1097/MEJ.0000000000000479 28639958

[144] Horiike T Nagai H Kitani S. Identification of Allergens in the Box Jellyfish Chironex Yamaguchii That Cause Sting Dermatitis. Int Arch Allergy Immunol (2015) 167(2):73–82. 10.1159/000434721 26201970

[145] Friedel N Scolnik D Adir D Glatstein M. Severe Anaphylactic Reaction to Mediterranean Jellyfish (Ropilhema Nomadica) Envenomation: Case Report. Toxicol Rep (2016) 3:427–9. 10.1016/j.toxrep.2016.03.006 PMC5615929 28959564

[146] Uri S Marina G Liubov G. Severe Delayed Cutaneous Reaction Due to Mediterranean Jellyfish (Rhopilema Nomadica) Envenomation. Contact Dermatitis (2005) 52(5):282–3. 10.1111/j.0105-1873.2005.00582.x 15899003

[147] Kubota S Nozawa A Yanai T Ozasa K Mori S Kurihara K. The Case of a 14-Year-Old Boy Who Experienced Anaphylaxis Due to Jellyfish (Mastigias Papua) Ingestion. Arerugi = [Allergy] (2017) 66(6):809–12. 10.15036/arerugi.66.809 28701647

[148] Cañas JA Rodrigo-Muñoz JM Rondon-Cepeda SH Bordehore C Fernández-Nieto M del Pozo V. Jellyfish Collagen: A New Allergen in the Beach. Ann Allergy Asthma Immunol (2018) 120(4):430–1. 10.1016/j.anai.2018.01.018 29410055

[149] Tibballs J Yanagihara AA Turner HC Winkel K. Immunological and Toxinological Responses to Jellyfish Stings. Inflammation Allergy Drug Targets (2011) 10(5):438–46. 10.2174/187152811797200650 PMC3773479 21824077

[150] Buttaravoli PMDF Leffler SMMDF. Marine Envenomations, 3rd ed. London: Elsevier Health Sciences (2012). pp. 574–80.

[151] Morabito R Marino A La Spada G. Nematocytes’ Activation in Pelagia Noctiluca (Cnidaria, Scyphozoa) Oral Arms. J Comp Physiol A (2012) 198(6):419–26. 10.1007/s00359-012-0720-7 22526110

[152] Varney SMMDFF. Bites and Stings, Sixth ed. Philaldelphia: Elsevier (2016). pp. 474–83.e1.

[153] Badré S. Bioactive Toxins From Stinging Jellyfish. Toxicon Off J Int Soc Toxinol (2014) 91:114–25. 10.1016/j.toxicon.2014.09.010 25286397

[154] Nagai H Takuwa K Nakao M Ito E Miyake M Noda M. Novel Proteinaceous Toxins From the Box Jellyfish (Sea Wasp) Carybdea Rastoni. Biochem Biophys Res Commun (2000) 275(2):582–8. 10.1006/bbrc.2000.3353 10964707

[155] Nagai H Takuwa K Nakao M Sakamoto B Crow GL Nakajima T. Isolation and Characterization of a Novel Protein Toxin From the Hawaiian Box Jellyfish (Sea Wasp) Carybdea Alata. Biochem Biophys Res Commun (2000) 275(2):589–94. 10.1006/bbrc.2000.3352 10964708

[156] Nagai H Takuwa-Kuroda K Nakao M Oshiro N Iwanaga S Nakajima T. A Novel Protein Toxin From the Deadly Box Jellyfish (Sea Wasp, Habu-Kurage) Chiropsalmus Quadrigatus. Biosci Biotechnol Biochem (2002) 66(1):97–102. 10.1271/bbb.66.97 11866126

[157] Brinkman DL Konstantakopoulos N McInerney BV Mulvenna J Seymour JE Isbister GK. Chironex Fleckeri (Box Jellyfish) Venom Proteins: Expansion of a Cnidarian Toxin Family That Elicits Variable Cytolytic and Cardiovascular Effects. J Biol Chem (2014) 289(8):4798–812. 10.1074/jbc.M113.534149 PMC3931041 24403082

[158] Kramp PL. Synopsis of the Medusae of the World. J Marine Biol Assoc U Kingdom (1961) 40:7–382. 10.1017/S0025315400007347

[159] Tibballs J. Australian Venomous Jellyfish, Envenomation Syndromes, Toxins and Therapy. Toxicon Off J Int Soc Toxinol (2006) 48(7):830–59. 10.1016/j.toxicon.2006.07.020 16928389

[160] White J. Venom. In: Payne-James J Byard RW, editors. Encyclopedia of Forensic and Legal Medicine, 2nd ed. Oxford: Elsevier (2016). p. 622–38.

[161] Fox JW. Venoms and Poisons From Marine Organisms. In: Goldman L Schafer AI, editors. Goldman’s Cecil Medicine, 4th ed. Philadelphia: W.B. Saunders (2012). p. 697–700.

[162] Brinchmann BC Bayat M Brøgger T Muttuvelu DV Tjønneland A Sigsgaard T. A Possible Role of Chitin in the Pathogenesis of Asthma and Allergy. Ann Agric Environ Med (2011) 18(1):7–12. 21736263

[163] Burton OT Zaccone P. The Potential Role of Chitin in Allergic Reactions. Trends Immunol (2007) 28(10):419–22. 10.1016/j.it.2007.08.005 17826333

[164] Lee CG Da Silva CA Dela Cruz CS Ahangari F Ma B Kang M-J. Role of Chitin and Chitinase/Chitinase-Like Proteins in Inflammation, Tissue Remodeling, and Injury. Annu Rev Physiol (2011) 73:479–501. 10.1146/annurev-physiol-012110-142250 21054166 PMC3864643

[165] Inomata N Chin K Aihara M. Anaphylaxis Caused by Ingesting Jellyfish in a Subject With Fermented Soybean Allergy: Possibility of Epicutaneous Sensitization to Poly-Gamma-Glutamic Acid by Jellyfish Stings. J Dermatol (2014) 41(8):752–3. 10.1111/1346-8138.12542 24943113

[166] Inomata N Miyakawa M Aihara M. Surfing as a Risk Factor for Sensitization to Poly(γ-Glutamic Acid) in Fermented Soybeans, Natto, Allergy. Allergology Int (2018) 67(3):341–6. 10.1016/j.alit.2017.11.001 29175280

[167] Shimabukuro-Vornhagen A Gödel P Subklewe M Stemmler HJ Schlößer HA Schlaak M. Cytokine Release Syndrome. J Immunother Cancer (2018) 6(1):56. 10.1186/s40425-018-0343-9 29907163 PMC6003181

[168] Suntharalingam G Perry MR Ward S Brett SJ Castello-Cortes A Brunner MD. Cytokine Storm in a Phase 1 Trial of the anti-CD28 Monoclonal Antibody TGN1412. New Engl J Med (2006) 355(10):1018–28. 10.1056/NEJMoa063842 16908486

[169] Wood AJ Darbyshire J. Injury to Research Volunteers; the Clinical-Research Nightmare. New Engl J Med (2006) 354(18):1869–71. 10.1056/NEJMp068082 16672696

[170] Reis MB Rodrigues FL Lautherbach N Kanashiro A Sorgi CA Alyne FGM. Interleukin-1 Receptor-Induced PGE2 Production Controls Acetylcholine-Mediated Cardiac Dysfunction and Mortality During Scorpion Envenomation. Nat Commun (2020) 11(1):5433–. 10.1038/s41467-020-19232-8 PMC7595177 33116136

[171] Tiong K. Irukandji Syndrome, Catecholamines, and Mid-Ventricular Stress Cardiomyopathy. Eur Heart J - Cardiovasc Imaging (2008) 10(2):334–6. 10.1093/ejechocard/jen246 18801721

[172] Petricevich VL. Scorpion Venom and the Inflammatory Response. Mediators Inflammation (2010) 2010:903295–. 10.1155/2010/903295 PMC2838227 20300540

[173] Santos MS Silva CG Neto BS Grangeiro Junior CR Lopes VH Teixeira Junior AG. Clinical and Epidemiological Aspects of Scorpionism in the World: A Systematic Review. Wilderness Environ Med (2016) 27(4):504–18. 10.1016/j.wem.2016.08.003 27912864

[174] Comellas AP Pesce LM Azzam Z Saldias FJ Sznajder JI. Scorpion Venom Decreases Lung Liquid Clearance in Rats. Am J Respir Crit Care Med (2003) 167(8):1064–7. 10.1164/rccm.200207-688OC 12684245

[175] Pessini AC de Souza AM Faccioli LH Gregório ZMO Arantes EC. Time Course of Acute-Phase Response Induced by Tityus Serrulatus Venom and TsTX-I in Mice. Int Immunopharmacol (2003) 3(5):765–74. 10.1016/S1567-5769(03)00078-X 12757745

[176] Santhosh KN Pavana D Thippeswamy NB. Impact of Scorpion venoWm as an Acute Stressor on the Neuroendocrine-Immunological Network. Toxicon Off J Int Soc Toxinol (2016) 122:113–8. 10.1016/j.toxicon.2016.09.021 27697428

[177] Megdad-Lamraoui A Adi-Bessalem S Laraba-Djebari F. Cerebrospinal Inflammatory Response Following Scorpion Envenomation: Role of Histamine H1 and H3 Receptors. Inflammopharmacology (2019) 27(3):589–601. 10.1007/s10787-018-00553-6 30604198

[178] Zoccal KF da SilvaBitencourt C CA S. (2013) 61(1):1–10.

[179] Muraoka N Nara K Tamura F Kojima H Yamakawa H Sadahiro T. Role of cyclooxygenase-2-mediated Prostaglandin E2-Prostaglandin E Receptor 4 Signaling in Cardiac Reprogramming. Nat Commun (2019) 10(1):674. 10.1038/s41467-019-08626-y 30787297 PMC6382796

[180] Zoccal KF Gardinassi LG AtérioSorgi C Meirelles AFG Bordon KCF Glezer I. CD36 Shunts Eicosanoid Metabolism to Repress CD14 Licensed interleukin-1beta Release and Inflammation. Front Immunol (2018) 9:890. 10.3389/fimmu.2018.00890 29755470 PMC5934479

[181] Mégarbane B Abroug F Soulaymani R Comellas AP Pesce LM Patil SN. Scorpion Envenomation. New Engl J Med (2014) 371(16):1557–60. 10.1056/NEJMc1410354 25317885

[182] Board on Health Sciences P, Institute of M, Committee on Conflict of Interest in Medical Research E, Practice. The Pathway From Idea to Regulatory Approval: Examples for Drug Development. Bernard L Marilyn JF, editors. Washington (DC): National Academies Press (2009). p. 375–83.

[183] Currie BJ Jacups SP. Prospective Study of Chironex Fleckeri and Other Box Jellyfish Stings in the “Top End” of Australia’s Northern Territory. Med J Aust (2005) 183(11-12):631–6. 10.5694/j.1326-5377.2005.tb00062.x 16336157

[184] Tibballs J Li R Tibballs HA Gershwin LA Winkel KD. Australian Carybdeid Jellyfish Causing “Irukandji Syndrome”. Toxicon Off J Int Soc Toxinol (2012) 59(6):617–25. 10.1016/j.toxicon.2012.01.006 22361384

[185] Fenner P Carney I. The Irukandji Syndrome. A Devastating Syndrome Caused by a North Australian Jellyfish. Australian Family Physician (1999) 28(11):1131–7. 10615756

[186] Huynh TT Pereira P Mulcahy R Cullen P Seymour J Carrette T. Severity of Irukandji Syndrome and Nematocyst Identification From Skin Scrapings. Med J Aust (2003) 178(1):38–41. 10.5694/j.1326-5377.2003.tb05041.x 12492390

[187] Barnes JH. Cause and Effect in Irukandji Stingings. Med J Aust (1964) 1:897–904. 10.5694/j.1326-5377.1964.tb114424.x 14172390

[188] Flecker H. Irukandji Sting to North Queensland Bathers Without Production of Weals But With Severe General Symptoms. Med J Aust (1952) 2(3):89–91. 10.5694/j.1326-5377.1952.tb100081.x 14956317

[189] Courtney R Browning S Seymour J. Early Life History of the ‘Irukandji’ Jellyfish Carukia Barnesi. PloS One (2016) 11(3):e0151197–e. 10.1371/journal.pone.0151197 PMC4783009 26954781

[190] Courtney R Sachlikidis N Jones R Seymour J. Prey Capture Ecology of the Cubozoan Carukia Barnesi. PloS One (2015) 10(5):e0124256. 10.1371/journal.pone.0124256 25970583 PMC4429964

[191] McIver LJMM Tjhung IGMD Parish STMDTM Derkenne RCBADTM Kippin ANMM. Irukandji Syndrome in the Torres Strait: A Series of 8 Cases. Wilderness Environ Med (2011) 22(4):338–42. 10.1016/j.wem.2011.08.002 22000547

[192] Tibballs J. Envenomation. In: Bersten A, Handy J, editors. Oh's Intensive Care Manual. Eighth ed. China: Elsevier (2019). pp. 1006–18.e2.

[193] Nickson CP Waugh EB Jacups SP Currie BJ. Irukandji Syndrome Case Series From Australia’s Tropical Northern Territory. Ann Emergency Med (2009) 54(3):395–403. 10.1016/j.annemergmed.2009.03.022 19409658

[194] Nickson CP Currie BJ Fenner PJ. Priapism and Irukandji Syndrome. Ann Emergency Med (2010) 55(6):581–2. 10.1016/j.annemergmed.2010.01.006 20494230

[195] Little M Pereira P Mulcahy R Cullen P Carrette T Seymour J. Severe Cardiac Failure Associated With Presumed Jellyfish. Sting Irukandji syndrome? Anaesthesia Intensive Care (2003) 31(6):642–7. 10.1177/0310057X0303100605 14719425

[196] Fenner PJ Williamson JA Gunawardane K Murtha W Burnett JW Colquhoun DM. The “Irukandji Syndrome” and Acute Pulmonary Oedema. Med J Aust (1988) 149(3):150–6. 10.5694/j.1326-5377.1988.tb120544.x 2899834

[197] Pereira P Barry J Corkeron M Keir P Little M Seymour J. Intracerebral Hemorrhage and Death After Envenoming by the Jellyfish Carukia Barnesi. Clin Toxicol (Philadelphia Pa) (2010) 48(4):390–2. 10.3109/15563651003662675 20507250

[198] Ramasamy S Isbister GK Seymour JE Hodgson WC. The In Vivo Cardiovascular Effects of the Irukandji Jellyfish (Carukia Barnesi) Nematocyst Venom and a Tentacle Extract in Rats. Toxicol Lett (2005) 155(1):135–41. 10.1016/j.toxlet.2004.09.004 15585368

[199] Bailey PM. Fatal Envenomation by Jellyfish Causing Irukandji Syndrome. Med J Aust (2003) 178(3):139; author reply –40. 10.5694/j.1326-5377.2003.tb05108.x 12558488

[200] Winkel KD Tibballs J Molenaar P Lambert G Coles P Ross-Smith M. Cardiovascular Actions of the Venom From the Irukandji (Carukia Barnesi) Jellyfish: Effects in Human, Rat and Guinea-Pig Tissues In Vitro and in Pigs In Vitro. Clin Exp Pharmacol Physiol (2005) 32(9):777–88. 10.1111/j.1440-1681.2005.04258.x 16173936

[201] Corkeron M Pereira P Makrocanis C. Early Experience With Magnesium Administration in Irukandji Syndrome. Anaesthesia Intensive Care (2004) 32(5):666–9. 10.1177/0310057X0403200510 15535491

[202] McCullagh N Pereira P Cullen P Mulcahy R Bonin R Little M. Randomised Trial of Magnesium in the Treatment of Irukandji Syndrome. Emergency Med Australasia EMA (2012) 24(5):560–5. 10.1111/j.1742-6723.2012.01602.x 23039299

[203] Rathbone J Franklin R Gibbs C Williams D. Review Article: Role of Magnesium Sulphate in the Management of Irukandji Syndrome: A Systematic Review. Emergency Med Australasia EMA (2017) 29(1):9–17. 10.1111/1742-6723.12694 27748058

[204] Sugimoto J Romani AM Valentin-Torres AM Luciano AA Ramirez Kitchen CM Funderburg N. Magnesium Decreases Inflammatory Cytokine Production: A Novel Innate Immunomodulatory Mechanism. J Immunol (Baltimore Md 1950) (2012) 188(12):6338–46. 10.4049/jimmunol.1101765 PMC3884513 22611240

[205] Siddall E Radhakrishnan J. Capillary Leak Syndrome: A Cytokine and Catecholamine Storm? Kidney Int (2019) 95(5):1009–11. 10.1016/j.kint.2019.03.001 31010471

[206] Staedtke V Bai RY Kim K Darvas M Davila ML Riggins GJ. Disruption of a Self-Amplifying Catecholamine Loop Reduces Cytokine Release Syndrome. Nature (2018) 564(7735):273–7. 10.1038/s41586-018-0774-y PMC6512810 30542164

[207] Hwang D-S Kim SK Bae H. Therapeutic Effects of Bee Venom on Immunological and Neurological Diseases. Toxins (2015) 7(7):2413–21. 10.3390/toxins7072413 PMC4516920 26131770

[208] Grgic I Wulff H Eichler I Flothmann C Kohler R Hoyer J. Blockade of T-lymphocyte Kca3.1 and Kv1.3 Channels as Novel Immunosuppression Strategy to Prevent Kidney Allograft Rejection. Transplant Proc (2009) 41(6):2601–6. 10.1016/j.transproceed.2009.06.025 PMC3343637 19715983

[209] Kou JQ Han R Xu YL Ding XL Wang SZ Chen CX. Differential Effects of Naja Naja Atra Venom on Immune Activity. Evidence-Based complementary Altern Med eCAM (2014) 2014:287631. 10.1155/2014/287631 PMC4082923 25024726

[210] Nam S Ko E Park SK Ko S Jun CY Shin MK. Bee Venom Modulates Murine Th1/Th2 Lineage Development. Int Immunopharmacol (2005) 5(9):1406–14. 10.1016/j.intimp.2005.03.011 15953567

[211] Xu YL Kou JQ Wang SZ Chen CX Qin ZH. Neurotoxin From Naja Naja Atra Venom Inhibits Skin Allograft Rejection in Rats. Int Immunopharmacol (2015) 28(1):188–98. 10.1016/j.intimp.2015.05.040 26071222

[212] Grafton G Thwaite L. Calcium Channels in Lymphocytes. Immunology (2001) 104(2):119–26. 10.1046/j.0019-2805.2001.01321.x PMC1783295 11683950

[213] Cahalan MD Wulff H Chandy KG. Molecular Properties and Physiological Roles of Ion Channels in the Immune System. J Clin Immunol (2001) 21(4):235–52. 10.1023/A:1010958907271 11506193

[214] Feske S Wulff H Skolnik EY. Ion Channels in Innate and Adaptive Immunity. Annu Rev Immunol (2015) 33:291–353. 10.1146/annurev-immunol-032414-112212 25861976 PMC4822408

[215] Hu L Wang T Gocke AR Nath A Zhang H Margolick JB. Blockade of Kv1.3 Potassium Channels Inhibits Differentiation and Granzyme B Secretion of Human CD8+ T Effector Memory Lymphocytes. PloS One (2013) 8(1):e54267. 10.1371/journal.pone.0054267 23382885 PMC3559683

[216] Schmalhofer WA Bao J McManus OB Green B Matyskiela M Wunderler D. Identification of a New Class of Inhibitors of the Voltage-Gated Potassium Channel, Kv1.3, With Immunosuppressant Properties. Biochemistry (2002) 41(24):7781–94. 10.1021/bi025722c 12056910

[217] Wulff H Calabresi PA Allie R Yun S Pennington M Beeton C. The Voltage-Gated Kv1.3 K(+) Channel in Effector Memory T Cells as New Target for MS. J Clin Invest (2003) 111(11):1703–13. 10.1172/JCI16921 PMC156104 12782673

[218] Alama A Bruzzo C Cavalieri Z Forlani A Utkin Y Casciano I. Inhibition of the Nicotinic Acetylcholine Receptors by Cobra Venom α-Neurotoxins: Is There a Perspective in Lung Cancer Treatment? PloS One (2011) 6(6):e20695–e. 10.1371/journal.pone.0020695 PMC3113800 21695184

[219] Zhao Y Huang J Yuan X Peng B Liu W Han S. Toxins Targeting the Kv1.3 Channel: Potential Immunomodulators for Autoimmune Diseases. Toxins (2015) 7(5):1749–64. 10.3390/toxins7051749 PMC4448172 25996605

[220] Koshy S Huq R Tanner MR Atik MA Porter PC Khan FS. Blocking KV1.3 Channels Inhibits Th2 Lymphocyte Function and Treats a Rat Model of Asthma. J Biol Chem (2014) 289(18):12623–32. 10.1074/jbc.M113.517037 PMC4007452 24644290

[221] Matheu MP Beeton C Garcia A Chi V Rangaraju S Safrina O. Imaging of Effector Memory T Cells During a Delayed-Type Hypersensitivity Reaction and Suppression by Kv1.3 Channel Block. Immunity (2008) 29(4):602–14. 10.1016/j.immuni.2008.07.015 PMC2732399 18835197

[222] Panyi G Possani LD Rodriguez de la Vega RC Gaspar R Varga Z. K+ Channel Blockers: Novel Tools to Inhibit T Cell Activation Leading to Specific Immunosuppression. Curr Pharm Design (2006) 12(18):2199–220. 10.2174/138161206777585120 16787250

[223] Toldi G Bajnok A Dobi D Kaposi A Kovács L Vásárhelyi B. The Effects of Kv1.3 and IKCa1 Potassium Channel Inhibition on Calcium Influx of Human Peripheral T Lymphocytes in Rheumatoid Arthritis. Immunobiology (2012) 218(3):311–6. 10.1016/j.imbio.2012.05.013 22705192

[224] Zhang S Wang X Ju C Zhu L Du Y Gao C. Blockage of K(Ca)3.1 and Kv1.3 Channels of the B Lymphocyte Decreases the Inflammatory Monocyte Chemotaxis. Int Immunopharmacol (2016) 31:266–71. 10.1016/j.intimp.2015.12.032 26795234

[225] Ali SA Alam M Abbasi A Kalbacher H Schaechinger TJ Hu Y. Structure–Activity Relationship of a Highly Selective Peptidyl Inhibitor of Kv1.3 Voltage-Gated K+-channel From Scorpion (B. Sindicus) Venom. Int J Pept Res Ther (2014) 20(1):19–32.

[226] Cahalan MD Chandy KG. The Functional Network of Ion Channels in T Lymphocytes. Immunol Rev (2009) 231(1):59–87. 19754890 10.1111/j.1600-065X.2009.00816.x PMC3133616

[227] Pennington MW Beeton C Galea CA Smith BJ Chi V Monaghan KP. Engineering a Stable and Selective Peptide Blocker of the Kv1.3 Channel in T Lymphocytes. Mol Pharmacol (2009) 75(4):762–73. 10.1124/mol.108.052704 PMC2684922 19122005

[228] Mouhat S Jouirou B Mosbah A De Waard M Sabatier J-M. Diversity of Folds in Animal Toxins Acting on Ion Channels. Biochem J (2004) 378(3):717–26. 10.1042/bj20031860 PMC1224033 14674883

[229] Herzig V King GF. The Cystine Knot is Responsible for the Exceptional Stability of the Insecticidal Spider Toxin ω-Hexatoxin-Hv1a. Toxins (2015) 7(10):4366–80. 10.3390/toxins7104366 PMC4626739 26516914

[230] Liu Y-L Lin H-M Zou R Wu J-C Han R Raymond LN. Suppression of Complete Freund’s Adjuvant-Induced Adjuvant Arthritis by Cobratoxin. Acta Pharmacol Sin (2009) 30(2):219–27. 10.1038/aps.2008.20 PMC4002463 19169271

[231] Choi MS Choi T Park S Lee G Haam K-K Hong M-C. Bee Venom Ameliorates Ovalbumin Induced Allergic Asthma Via Modulating CD4+CD25+ Regulatory T Cells in Mice. Cytokine (2013) 61(1):256–65. 10.1016/j.cyto.2012.10.005 23121887

[232] Elias JA Lee CG Zheng T Ma B Homer RJ Zhu Z. New Insights Into the Pathogenesis of Asthma. J Clin Invest (2003) 111(3):291–7. 10.1172/JCI17748 PMC151878 12569150

[233] Gilmore TD. Introduction to NF-kappaB: Players, Pathways, Perspectives. Oncogene (2006) 25(51):6680. 10.1038/sj.onc.1209954 17072321

[234] Gu SM Park MH Hwang CJ Song HS Lee US Han SB. Bee Venom Ameliorates Lipopolysaccharide-Induced Memory Loss by Preventing NF-kappaB Pathway. J Neuroinflamm (2015) 12(1):124. 10.1186/s12974-015-0344-2 PMC4501073 26112466

[235] Lawrence T. The Nuclear Factor NF-Kappa B Pathway in Inflammation. Cold Spring Harbor Perspect Biol (2009) 1(6):a001651. 10.1101/cshperspect.a001651 PMC2882124 20457564

[236] Palm Noah W Rosenstein Rachel K Yu S Schenten Dominik D Florsheim E Medzhitov R. Bee Venom Phospholipase A2 Induces a Primary Type 2 Response That is Dependent on the Receptor ST2 and Confers Protective Immunity. Immunity (2013) 39(5):976–85. 10.1016/j.immuni.2013.10.006 PMC3852615 24210353

[237] Garg R Juncadella IJ Ramamoorthi N Ashish Ananthanarayanan SK Thomas V. Cutting Edge: CD4 is the Receptor for the Tick Saliva Immunosuppressor, Salp15. J Immunol (2006) 177(10):6579–83. 10.4049/jimmunol.177.10.6579 PMC4302324 17082567

[238] Zhang M Caragine T Wang H Cohen PS Botchkina G Soda K. Spermine Inhibits Proinflammatory Cytokine Synthesis in Human Mononuclear Cells: A Counterregulatory Mechanism That Restrains the Immune Response. J Exp Med (1997) 185(10):1759–68. 10.1084/jem.185.10.1759 PMC2196317 9151701

